# Clinical Effectiveness of AI-Based Cardiac Arrest Prediction System for General Ward Patients (VITAL-MD): Protocol for a Multicenter Pragmatic Stepped Wedge Cluster Randomized Controlled Trial

**DOI:** 10.2196/104230

**Published:** 2026-10-19

**Authors:** Mincheol Kim, Kyung Hoon Kim, Minsoo Kim, Sun Jung Kwon, Myung Soo Park, Ah Jin Kim, Man-Jong Lee, Mi Hwa Park, Hyung Keun Cha, Minhyeok Lee, In Beom Jeong, Soon Joo Wang, Sung Bak An, Jeen Hwa Lee, Kyung-Jae Cho, Jinhui Jang, HyoJin Jeong, Yoon Young Lee, Woori Han, VITAL-MD Study Group, Jung Soo Kim, Dongjoon Yoo

**Affiliations:** 1 VUNO, Seoul, Republic of Korea; 2 Department of Digital Health, Samsung Advanced Institute for Health Sciences & Technology, Sungkyunkwan University, Seoul, Republic of Korea; 3 Department of Internal Medicine, Division of Pulmonary and Critical Care Medicine, College of Medicine, Incheon St. Mary's Hospital, The Catholic University of Korea, Incheon, Republic of Korea; 4 Department of Internal Medicine, Division of Pulmonology and Critical Care Medicine, College of Medicine, Konyang University Hospital, Konyang University, Daejeon, Republic of Korea; 5 Department of Cardiology, College of Medicine, Dongtan Sacred Heart Hospital, Hallym University, Hwaseong, Gyeonggi-do, Republic of Korea; 6 Department of Critical Care Medicine, Inha University Hospital, 27 Inhang-ro, Jung-gu, Incheon, 22332, Republic of Korea, 82 32-890-2114, 82 32-890-2114; 7 Department of Emergency Medicine, College of Medicine, Dongtan Sacred Heart Hospital, Hallym University, Hwaseong, Republic of Korea; 8 Department of General Surgery, College of Medicine, Dongtan Sacred Heart Hospital, Hallym University, Hwaseong, Republic of Korea; 9 Department of Hospital Medicine, Critical Care Support Part, Inha University Hospital, Incheon, Republic of Korea; 10 Department of Nursing, Rapid Response Team, Konyang University Hospital, Daejeon, Republic of Korea; 11 Department of Nursing, Incheon St. Mary's Hospital, The Catholic University of Korea, Incheon, Republic of Korea; 12 Rapid Response Team, Dongtan Sacred Heart Hospital, Hwaseong, Republic of Korea

**Keywords:** rapid response system, rapid response team, early warning score, artificial intelligence, deep learning, clinical deterioration, clinical decision support systems, patient-centered outcome, randomized controlled trial, in-hospital cardiac arrest

## Abstract

**Background:**

In-hospital cardiac arrest (IHCA) remains a frequent and devastating event, and conventional early warning scores (EWSs) coupled with rapid response systems (RRSs) have shown inconsistent effects on patient-centered outcomes, largely owing to limited predictive accuracy and high false-alarm burden. AI-based EWSs (AI-EWSs) have demonstrated superior discrimination retrospectively, but high-quality prospective evidence in true external validation environments remains scarce. Existing AI-EWS trials have either been confined to internal-development settings or bundled the algorithm with specific implementation infrastructure, leaving the stand-alone effect of AI-based software as a medical device (AI-SaMD) on patient outcomes uncharacterized.

**Objective:**

This trial will evaluate whether implementation of AI-SaMD (VUNO Med-DeepCARS) as a stand-alone clinical decision-support tool in routine general-ward care reduces IHCA compared with usual care based on conventional EWSs.

**Methods:**

The VITAL-MD trial (validation of an AI-based clinical deterioration prediction model on patient outcomes through a stepped-wedge cluster randomized trial [SW-CRT] multicenter evaluation of DeepCARS) is a pragmatic, multicenter, SW-CRT conducted at 4 tertiary academic hospitals in the Republic of Korea. Six tertiary academic hospitals were assessed for eligibility based on inpatient capacity, RRS operating hours aligned with the national RRS pilot program, and no prior AI-SaMD use. All adult patients aged 19 years or older admitted to general wards during the 12-month trial period are included; patients younger than 19 years, or with no documented vital signs throughout hospitalization, are excluded. The 4 sites are randomized to sequentially transition from preimplementation (usual care) to postimplementation (AI-SaMD–guided care) in 4 steps separated by 7-day washout periods. DeepCARS generates real-time risk scores from 4 vital signs and integrates into the existing electronic medical record interface without altering RRS workflows. The primary outcome is the incidence of general-ward IHCA, defined per the Utstein guidelines, among the target cohort (defined as patients exceeding the alert threshold at least once). Assuming a baseline IHCA incidence of 6.70%, a 40% relative risk reduction, an intracluster correlation coefficient of 0.01, and 350 patients per cluster-period, 7002 target-cohort patients (total enrollment approximately 72,695) provide 80% power. Analyses follow the intention-to-treat principle using generalized linear mixed models adjusting for prespecified covariates, with prespecified sensitivity, subgroup, and compliance analyses.

**Results:**

The trial was registered with the Clinical Research Information Service (KCT0010243) on February 28, 2025. Recruitment was conducted from April 1, 2025, to March 31, 2026. All 4 sites completed transition to the postimplementation period, and data cleaning is currently underway before the final database lock, with primary results expected in 2027.

**Conclusions:**

VITAL-MD will provide the first large-scale, multicenter randomized evidence evaluating whether an AI-EWS, deployed as a stand-alone device in external validation environments and benchmarked against real-world standard-of-care practice, improves patient-centered outcomes for general-ward patients, advancing the evaluation of AI-SaMD from retrospective diagnostic accuracy toward prospective, action-anchored clinical utility.

## Introduction

In-hospital cardiac arrest (IHCA) remains a frequent and devastating event among hospitalized patients. In the United States alone, approximately 292,000 patients experience IHCA annually [1]. Globally, the reported incidence in adults ranges from 1.2 to 10 cases per 1000 admissions, and although survival to hospital discharge has improved over time, long-term prognosis remains poor; a systematic review and meta-analysis estimated 1-year survival after IHCA at 13.4% [2].

Historically, hospital patient safety initiatives focused predominantly on the “efferent limb” of the response, optimizing code teams to react to arrests. Over the past 2 decades, the paradigm has shifted toward the “afferent limb,” emphasizing the proactive detection and prevention of clinical deterioration through early warning scores (EWS) and rapid response systems (RRSs) [3]. Multiple national and international bodies, including the American Heart Association (AHA) and the Society of Critical Care Medicine (SCCM), strongly recommend EWSs and hospital-wide surveillance to trigger RRS interventions in general wards [4-7].

Despite these broad recommendations, the evidence supporting the implementation of conventional EWSs and RRSs in improving patient-centered outcomes remains limited and inconsistent. The latest AHA guidelines highlight a stark paucity of high-quality adult data, relying primarily on pediatric randomized controlled trials (RCTs) to substantiate EWS and RRS recommendations [4]. Furthermore, a recent Cochrane systematic review concluded that implementing conventional EWS and RRS on adult hospital wards may make little to no difference in key outcomes, such as IHCA incidence and in-hospital mortality [8]. A primary driver of this inconsistency is the suboptimal predictive accuracy of conventional track-and-trigger EWS tools, such as the National Early Warning Score 2 (NEWS2) and Modified Early Warning Score (MEWS). These scoring systems often have low sensitivity and positive predictive value, generating excessive numbers of nonactionable false-positive alerts. This overwhelms clinicians, causes alarm fatigue, and misdirects scarce critical care resources, while true episodes of atypical deterioration may still go unrecognized [9,10].

To overcome these critical limitations, AI and machine learning (ML) models have been actively developed. Retrospectively, AI-based EWSs consistently demonstrate superior discrimination capabilities (eg, area under the receiver operating characteristic curve [AUROC]) compared to conventional scores. However, exceptional algorithmic performance does not automatically translate into clinical utility, as emphasized by the Developmental and Exploratory Clinical Investigations of Decision Support Systems Driven by AI (DECIDE-AI) guidelines and recent statements from the AHA and the *Journal of the American Medical Association (JAMA)*, calling for evaluations using robust, patient-centered end points [11-13].

Indeed, the prospective clinical effectiveness of AI-based EWSs (AI-EWSs) in RCTs remains uncertain. For instance, the Continuous Monitoring of Event Trajectories (CoMET) pragmatic RCT reported negative results, with a passive visual display of AI-driven clinical deterioration risk failing to improve patient outcomes [14]. Conversely, the Communicating Narrative Concerns Entered by Registered Nurses (CONCERN) trial demonstrated significant reductions in mortality; however, its evaluation was conducted largely within the internal development environment [15]. As highlighted by recent analyses, deep learning models often experience significant cross-site performance degradation when deployed in external environments, raising substantial concerns regarding their generalizability and the need for additional local retraining to meet the promised performance [16,17]. For example, an external validation study of Pacmed (Pacmed B V)—a commercial AI device that obtained European regulatory clearance—revealed that the model experienced severe performance degradation when deployed at an independent site, ultimately requiring retraining to restore the intended predictive accuracy [18].

The AI-based software as a medical device (AI-SaMD) used in this study, VUNO Med-DeepCARS (version 1.3.2), continuously predicts the risk of IHCA within 24 hours using only 4 routinely measured vital signs. Unlike models that struggle with generalizability, DeepCARS has demonstrated robustness and discriminative accuracy across diverse clinical settings. Following its initial development [19], its clinical utility has been extensively validated through single-center and large-scale multicenter retrospective cohorts [20,21], as well as a prospective multicenter observational study [22]. Furthermore, real-world external validations have confirmed its efficacy during both RRS operating and nonoperating periods [23] and highlighted its practical utility during severe national health care workforce shortages [24]. Most notably, its implementation was associated with a 35% reduction in in-hospital mortality in a recent nonrandomized controlled trial (NRCT) [25].

We therefore designed the present study, the VITAL-MD trial (validation of an AI-based clinical deterioration prediction model on patient outcomes through a stepped-wedge cluster randomized trial [SW-CRT]: a multicenter evaluation of DeepCARS). To our knowledge, VITAL-MD is the first large-scale multicenter randomized trial designed to evaluate an AI-EWS as a stand-alone device in external validation environments, addressing the 2 principal evidence gaps in the existing AI-EWS literature. This study aims to rigorously evaluate whether the implementation of an AI-SaMD into routine clinical workflows improves patient-centered outcomes, thereby establishing a milestone for the prospective validation of medical AI.

## Methods

### Overview

This study was structured according to the recommendations of the SPIRIT (Standard Protocol Items: Recommendations for Interventional Trials) 2025 statement (Checklist 1).

### Objectives

The primary objective of this trial is to evaluate whether implementation of an AI-SaMD as a stand-alone clinical decision-support tool in routine general-ward care reduces the incidence of IHCA compared with usual care based on conventional EWSs, especially among adult patients identified as high-risk (target cohort).

Secondary objectives include evaluating the effect of the intervention on all-cause in-hospital mortality, hospital and intensive care unit (ICU) length of stay (LOS), time to unplanned ICU transfer after the first alert, and neurologic outcome at discharge among patients who experienced IHCA. A further objective is to characterize the clinical actions (eg, critical care consultation, antibiotic escalation, early ICU transfer) that may mediate any observed outcome benefit in order to translate algorithmic risk prediction into action-anchored clinical utility.

### Estimand Framework

The primary estimand will follow a treatment-policy strategy: the population-level effect of being admitted to a cluster operating in the postimplementation (AI-SaMD-guided) phase versus the preimplementation (usual care) phase, regardless of individual clinician adherence to AI-SaMD alerts, with the outcome variable defined as the binary occurrence of general-ward IHCA during hospitalization.

### Trial Design

This study is designed as a pragmatic, multicenter SW-CRT [26]. The unit of randomization is the hospital (cluster); the 4 participating hospitals were randomized to sequentially transition from control to intervention according to a prespecified schedule: preimplementation, transitional (washout), and postimplementation periods (Figure 1). This SW-CRT design allows each cluster to serve as its own control, accounting for intercluster variability. The total study period is 1 year to ensure coverage of all seasonal variations.

**Figure 1.**
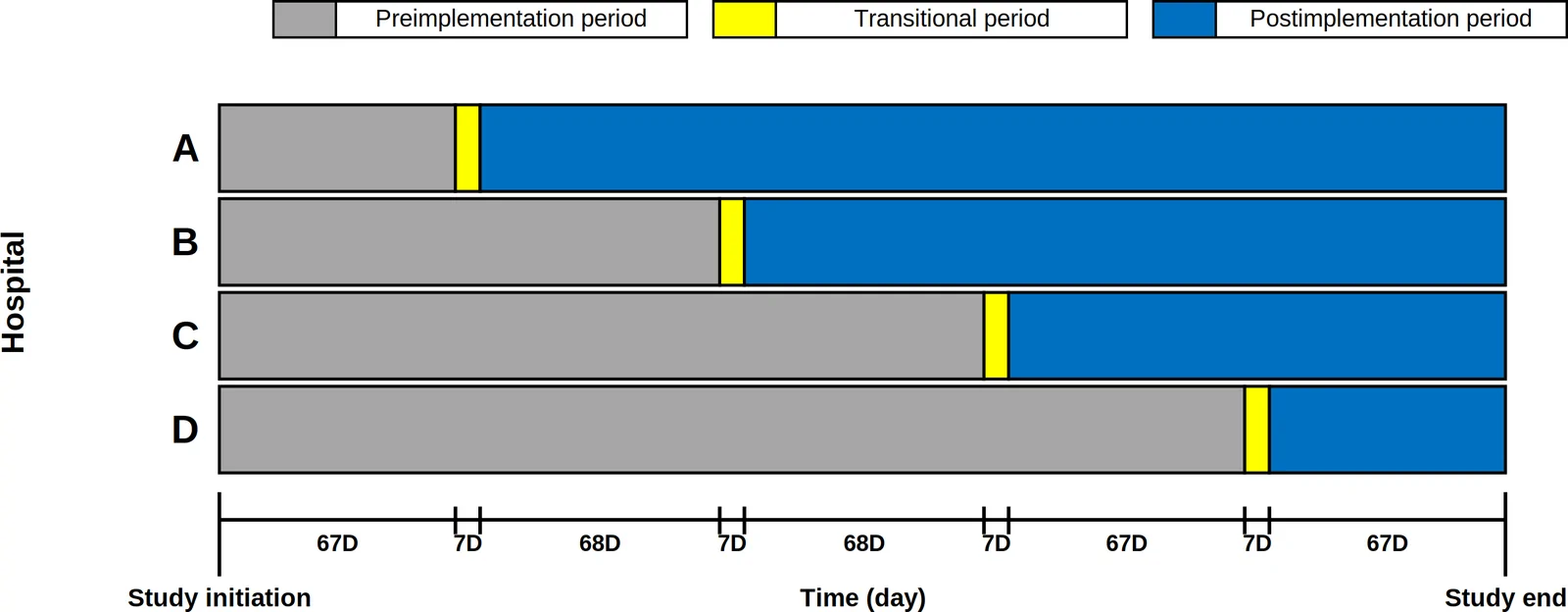
Schematic diagram of the stepped-wedge randomized design. The diagram illustrates the sequence of preimplementation, transitional (washout), and postimplementation periods. Each wedge spans 2.4 months and includes a 7-day transitional period.

The detailed study design is presented in Figure 2. Intervention effectiveness will be evaluated by comparing patient outcomes between the AI-SaMD-guided (postimplementation) and usual care (preimplementation) phases. Within this overall comparison, the primary analysis will focus on the target cohort, defined as patients who exceed the AI-SaMD alert threshold at least once during general-ward hospitalization. This cohort aligns with the intended use of AI-SaMD (DeepCARS), which is designed to identify patients at high risk of clinical deterioration, and with the clinical estimand of interest, as an RRS is by definition intended to identify patients at risk of deterioration and facilitate intervention [3,19,21-23,25,27]. Patients who do not exceed the alert threshold will be defined as the nontarget cohort. In prior studies, these non-high-risk patients demonstrated a very low incidence of the primary outcome [25,28-34]; the target cohort therefore represents the population in which the intervention is intended to act and in which a treatment effect can meaningfully be detected, supporting its designation as the primary analysis population. To validate the restriction of primary analysis to the target cohort, baseline characteristics, alert distribution, and patient outcomes will also be compared between the target and nontarget cohorts, as well as between the pre- and postimplementation phases, in a secondary analysis.

**Figure 2.**
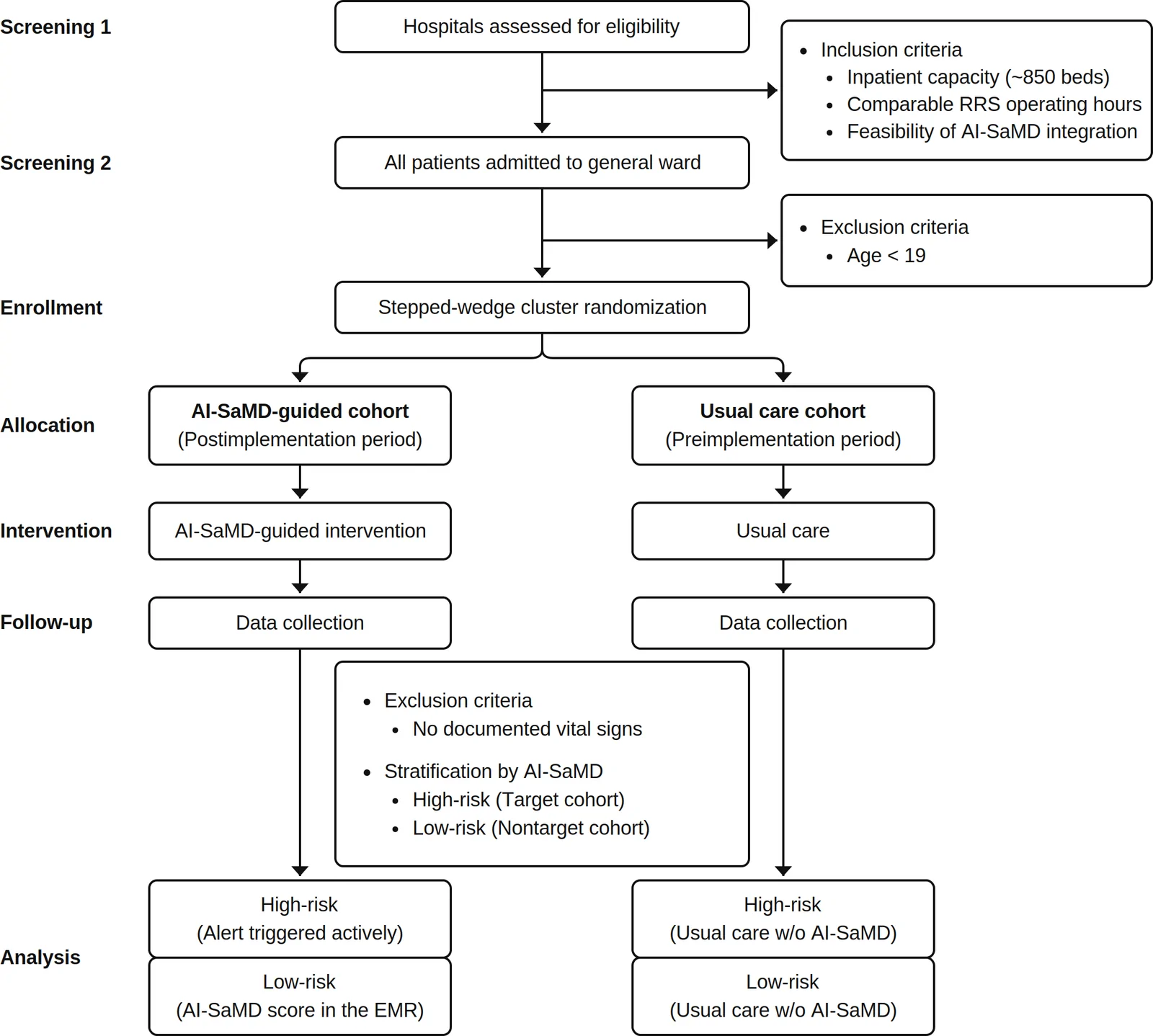
Trial flow diagram of the VITAL-MD stepped-wedge cluster randomized trial. AI-SaMD: AI-based software as a medical device; EMR: electronic medical record; RRS: rapid response system; VITAL-MD: validation of an AI-based clinical deterioration prediction model on patient outcomes through a stepped-wedge cluster randomized trial: multicenter evaluation of DeepCARS.

### Participants, Interventions, and Outcomes

#### Trial Setting

This multicenter trial will be conducted at tertiary academic hospitals across different regions in the Republic of Korea: Inha University Hospital, Hallym University Dongtan Sacred Heart Hospital, Konyang University Hospital, and The Catholic University of Korea Incheon St. Mary’s Hospital. All participating sites operate as inpatient tertiary care institutions with established RRS, and the AI-SaMD will be deployed within the general ward setting of each site.

#### Eligibility Criteria

Participating sites are selected based on the following criteria: (1) similar inpatient capacity (approximately 850 beds) to minimize intercluster heterogeneity; (2) comparable RRS operating hours, consistent with the national RRS pilot program [3,35-38]; and (3) no prior use of the AI-SaMD (DeepCARS), which will be newly integrated into the electronic medical record (EMR) system for the purpose of this trial. These criteria were selected to minimize structural heterogeneity across participating hospitals, thereby reducing unnecessary between-cluster variability. Of the 6 institutions assessed for eligibility, 4 were enrolled; 2 were excluded because RRSs were discontinued due to institutional resource reallocation in response to financial constraints.

All adult patients aged 19 years or older admitted to the general ward at participating hospitals during the trial period will be included. Consistent with the cluster-level allocation, patients are not individually recruited, sampled, or consented; the cohort reflects the full population of ward admissions during each exposure period. Exclusion criteria are patients younger than 19 years at admission or no documented vital signs throughout hospitalization, resulting in no AI-SaMD score generation.

#### Intervention and Comparator

##### Intervention Description

VUNO Med-DeepCARS (version 1.3.2) is a deep learning–based cardiac arrest risk management system in general-ward patients [19,21-23,25,27]. It generates real-time 0‐100 risk scores estimating the likelihood of cardiac arrest within 24 hours based on 4 routinely monitored vital signs (heart rate, respiratory rate, blood pressure, and body temperature), using the longitudinal measurements collected since hospital admission.

The AI-SaMD (DeepCARS) will be integrated into the EMR vital sign interface, replacing the conventional EWSs for patient monitoring and risk stratification without altering existing workflows. The risk score is updated whenever new vital signs are recorded, and alerts are generated when a predefined threshold is exceeded. To minimize alarm fatigue, hospital-specific thresholds were selected to achieve an alert volume comparable to that of the existing RRS, based on historical data from each site, and repeated alerts were suppressed within the same calendar day [21-23,25,27]. Upon alert activation, the RRS team reviews the patient and health care professionals (HCPs) are recommended to reassess care; however, no mandatory response is required. RRS operations and reassessment criteria follow the national RRS pilot program guidelines (Table 1) [35,36]. The AI-SaMD functions autonomously within routine workflows, preserving physician discretion and requiring no additional staffing or protocol modifications.

**Table 1.** List of reasons and types of interventions implemented in response to the AI-SaMD^a^ alert.

Reasons for intervention	Types of intervention
Respiratory distress	Treatment plan and consultation
Sepsis	Advice for referral consultation
Septic shock	Health care professional education
Hypotension	Patient/guardian education
Cardiac arrest	Consultation through telephone
Cardiogenic shock	ABGA^b^ Blood test POCUS^c^
Anaphylactic shock	Chest X-ray
Hypovolemic shock	EPOC (sort of ABGA)
Obstructive shock	EtCO2 monitoring^d^
Arrhythmia	Intubation
Metabolic acidosis	Ventilator
Altered mental status	Suction
For education/procedure	Low/high-flow oxygen supply
For transfer	Central line insertion
Others	Peripheral line insertion IV^e^ fluid and medication CPR^f^ DNR^g^ recommend UIT^h^ recommend

a AI-SaMD: AI-software as a medical device.

b ABGA: arterial blood gas analysis.

c POCUS: point-of-care ultrasound.

d EtCO2: end-tidal carbon dioxide.

e IV: intravenous.

f CPR: cardiopulmonary resuscitation.

g DNR: do not resuscitate.

h UIT: unplanned intensive care unit transfer.

##### Explanation for the Choice of Comparators

In the preimplementation period of the SW-CRT, usual care without the AI-SaMD serves as the comparator. Risk stratification relies on conventional EWSs as routinely deployed at each participating hospital, and RRS activation and subsequent response follow the national RRS pilot program in the Republic of Korea [3,35-38].

Usual care and the recent standard of care, rather than a sham device or no-monitoring comparator, were chosen for both ethical and methodological reasons. Conventional EWS-based monitoring and RRS activation are the current standard of care for detecting clinical deterioration in general-ward inpatients, as endorsed by the AHA and SCCM [4,5]. Withholding such monitoring from at-risk inpatients would violate clinical equipoise and the ethical principles of trials in acutely ill populations.

Methodologically, recent reviews and consensus statements have highlighted these considerations for the design of AI-EWS interventional studies. A systematic review of randomized clinical trials of ML interventions in *JAMA Network Open* reported that few such RCTs have been conducted, with high variability in adherence to reporting standards and substantial risk of bias across the existing evidence base [39]. The recent AHA Science Advisory on pragmatic approaches to health care AI evaluation has emphasized that AI tools should be evaluated in real-world contexts and against the care they would actually displace, rather than against artificially standardized comparators [40]. By using a routine established standard of care as the comparator, VITAL-MD estimates the added value of AI-SaMD over the care patients would otherwise receive.

This setting better reflects the question this trial seeks to answer, whether adding an AI-driven decision-support layer improves outcomes beyond what current EWS-anchored RRS systems already achieve. Although structural heterogeneity across participating hospitals was minimized through the site-selection criteria, real-world variation in day-to-day clinical workflows, staffing, and patient severity remained. By evaluating the AI-SaMD against usual care under these routine clinical conditions, the trial estimates the incremental benefit of the intervention in the setting in which it is intended to be used. These operational variations support the pragmatic generalizability of the trial.

### Criteria for Discontinuing or Modifying Allocated Interventions

No predefined criteria exist for discontinuing or modifying the intervention, as the AI-SaMD will operate passively within routine workflows and poses minimal risk.

Patients admitted during the preimplementation period who remain hospitalized during the postimplementation period will be considered crossover cases and analyzed separately [26].

### Strategies to Improve Adherence to Interventions

Adherence may be limited by delayed alert acknowledgment, noncompliance with alerts, patient refusal, alert fatigue, and staffing constraints [36,41-45].

To address these challenges, each participating hospital will undertake structured preimplementation preparation before AI-SaMD deployment. This will include (1) early coordination with hospital IT teams to enable seamless integration of the AI-SaMD into existing EMR and clinical workflows and (2) preimplementation education programs for relevant HCPs—including rapid response team members, ward nurses, and attending physicians—to standardize the interpretation of AI-SaMD outputs and the corresponding alert response procedures.

Ongoing education throughout the trial will further reinforce appropriate use and timely response to alerts. However, because the AI-SaMD-guided intervention is nonmandatory in order to preserve physician autonomy, full compliance cannot be guaranteed and will be evaluated in a secondary analysis.

### Relevant Concomitant Care Permitted or Prohibited During the Trial

All usual clinical practices are permitted. The AI-SaMD provides advisory alerts only and does not mandate clinical decisions.

### Patient and Public Involvement

No patient or public involvement was incorporated into the design or conduct of this trial, given the cluster-level intervention, waiver of individual consent, and exclusive reliance on routinely collected data.

### Outcomes

The primary outcome is the incidence of general-ward cardiac arrest, defined according to the in-hospital Utstein guidelines as cessation of cardiac activity with the absence of pulse, unresponsiveness, and apnea requiring resuscitation [46]. Secondary outcomes include all-cause in-hospital mortality, hospital and ICU LOS, time to unplanned ICU transfer (UIT) after the first alert, and Cerebral Performance Category (CPC) at discharge among general-ward patients with cardiac arrest. UIT is defined as ICU transfer within 24 hours for clinical deterioration, excluding planned postoperative ICU admissions [47].

### Harms

This study poses minimal risk, as the intervention (AI-SaMD) functions as an additional safety measure without altering usual care. Adverse events are expected to be rare and likely reflect underlying illness rather than the intervention. Adverse events will be recorded in the case report form (CRF) and reviewed during interim analyses by the Data and Safety Monitoring Board (DSMB) and institutional review board (IRB) at each participating site.

In addition, the conduct of this trial will be reported with the routine mortality and cardiac arrest conferences held at each of the 4 participating hospitals. If any case is judged at such a conference to raise concern about potential harm related to the intervention—for example, workflow disruption associated with the intervention—the conference participants may independently notify the site principal investigator (PI) and the DSMB chair at any time by telephone and email, using the contact details provided in the DSMB charter (Multimedia Appendix 1). Similarly, any notable increase in mortality or IHCA at a participating site that is considered at such a conference to be associated with AI-SaMD implementation may also be reported through the same channel at any time.

### Participant Timeline

The study schedule is shown in Table 2.

**Table 2.** Schedule of procedures during study period.

Procedure	Study period		
	Admission	General ward stay	Discharge
Screening/enrollment/allocation			
Eligibility screening	✓	—^a^	—
Exclusion criteria^b^	✓	—	✓
Randomization	✓	—	—
Intervention			
Intervention (AI-SaMD)^c^	—	✓	—
Comparator (usual care)	—	✓	—
Data collection^d^			
Baseline characteristics	✓	—	—
Daily vital signs	—	✓	—
Intervention records	—	✓	—
General ward cardiac arrest	—	—	✓
All-cause in-hospital mortality	—	—	✓
Hospital length of stay	—	—	✓
Total ICU^e^ length of stay	—	—	✓
Time to UIT^f^ after the first alert	—	—	✓
Cerebral Performance Category	—	—	✓

a Not applicable.

b Exclusion criteria: two-stage, patient-level age <19 years at admission; no vital-sign measurements recorded throughout hospitalization, resulting in no AI-based software as a medical device score being generated.

c AI-SaMD: AI-based software as a medical device.

d Data collection: vital signs and intervention records captured daily during ward stay; outcomes recorded from electronic medical record after discharge.

e ICU: intensive care unit.

f UIT: unplanned intensive care unit transfer.

### Sample Size

The required sample size is estimated to detect a reduction in general ward cardiac arrest among patients identified as high risk by the AI-SaMD (target cohort). The baseline incidence in the control group is assumed to be 6.70% based on prior studies [23,25]. Assuming a 40% relative risk reduction (RRR), consistent with previous trials in comparable high-risk populations, the expected incidence in the intervention group is set at 4.00% [25,28,29,48].

With a 2-sided α of .05, 80% power, an intracluster correlation coefficient (ICC) of 0.01 (based on a meta-analysis of RRS studies) [49], and 350 patients per cluster-period (derived from prior institutional data) [21-23], the required sample size is calculated at 7002 patients in the target cohort [50]. Assuming a 10% high-risk alert rate and 5% dropout, total enrollment is estimated at 72,695 [21-23], including 65,693 patients in the nontarget cohort.

### Recruitment

No specific recruitment strategy is required, as this study implements hospital-level intervention and includes all general ward admissions eligible for screening.

### Assignment of Interventions

#### Randomization: Sequence Generation

The transition order of the 4 participating hospitals was determined before study initiation through a centralized randomization procedure performed by an independent statistician not involved in trial conduct. Given the small number of clusters and the prespecified eligibility criteria ensuring baseline comparability across hospitals, no stratification was applied. Full procedural details are provided in Multimedia Appendix 2.

#### Allocation Concealment Mechanism

The randomization sequence for cluster transition order was generated by independent statisticians from a statistical contract research organization (CRO) and concealed from all site investigators, clinical staff, and the trial management group (TMG) until each cluster’s prescheduled notification. Participating hospitals will be notified of their assigned transition time point one month before the scheduled transition to allow for technical preparation.

#### Implementation

In this SW-CRT, clusters will be randomly assigned to transition across 4 sequential steps, dividing the 12-month study period into five 2.4-month phases. Patients admitted in the preimplementation period will receive usual care, and those admitted in the postimplementation period will receive AI-SaMD-guided intervention. All adult patients admitted to general wards at each participating hospital during the study period will be identified through routine EMR data extraction; no individual-level sampling, selective recruitment, or consent-based enrollment will be performed, consistent with the cluster-level design of the trial.

Patients hospitalized across both periods will be considered crossover cases [26]. To minimize contamination at each cluster’s phase transition, a 7-day transitional (washout) period, based on average general ward LOS, will be applied between the pre- and postimplementation periods [22,26,51].

#### Blinding

Given the stepped-wedge cluster deployment of an AI-SaMD, conventional blinding is not applicable, and detailed blinding is described here by role. Patients and caregivers are not informed of their site’s group assignment, as AI-SaMD outputs are restricted to an EMR interface with no patient access during both the pre- and postimplementation periods. HCPs and RRS staff are not blinded due to direct interaction with the AI-SaMD alerts, and hospitals are informed of their assigned transition one month before implementation; however, HCPs and RRS staff cannot access the AI-SaMD scores during the preimplementation period.

Statistical analyses, including interim analyses, will be conducted by independent statisticians at the CRO who receive intervention/control labels in masked form, with unblinding only after the primary analysis dataset is locked and approved by the principal investigator.

### Data Collection, Management, and Analysis

#### Data Collection Methods

Outcome data will be captured for all eligible patients admitted to participating general wards during the 12-month SW-CRT study period, irrespective of the cluster allocation phase.

Only routinely collected data and data from the national RRS pilot program will be used [35,36]. These include demographics (age, sex, admission and discharge dates, department, and ward information), routinely recorded vital signs (blood pressure, heart rate, respiratory rate, and body temperature), oxygen therapy (whether administered, type of equipment, and oxygen amount), oxygen saturation (SpO_2_), and Sequential Organ Failure Assessment (SOFA) components, along with the time of measurement. Baseline illness severity will be quantified by the admission SOFA score, computed from the first available measurements of each component during the index hospitalization, with per-component data sources adapted for general ward EMR availability, the preadmission carry-forward window for laboratory values, and handling of residual missingness operationalized consistently with prior work [52,53] and detailed in Multimedia Appendix 3. In addition, ICU transfers, cardiopulmonary resuscitation (CPR), do-not-resuscitate (DNR) orders, deaths, interventions, staffing, bed occupancy, and Charlson Comorbidity Index (CCI) components will be collected.

At each hospital, a site-level clinical research coordinator (CRC) will perform data collection and quality control. Data elements will be extracted from each participating hospital’s EMR and from its RRS module, which is built upon the national RRS pilot program [35,36]. The standardized structured data collection template, derived from the national RRS pilot program registry, will be deployed across all participating sites.

AI-SaMD scores will be stored in a separate EMR-based research module accessible only to the CRC for data collection and target/nontarget cohort allocation. The scores will not be visible to clinicians or guide clinical care during the preimplementation period. After transition to the postimplementation period, the AI-SaMD will be linked to the RRS module and integrated into the RRS workflow.

As the intervention is delivered at the cluster (hospital) level and outcomes are ascertained from routinely captured clinical data during the hospitalization period, individual-level retention strategies and follow-up adherence procedures are not applicable to this trial design.

#### Data Management

Data management will be conducted under the oversight of the PI in accordance with the Declaration of Helsinki and ICH-GCP guidelines.

Following site-level data entry and verification, each participating hospital will deidentify the captured records in conformity with the Health Insurance Portability and Accountability Act (HIPAA) deidentification standard and securely transfer them, via encrypted channels, to the designated trial server. The trial database is hosted on secure, access-controlled servers operated by VUNO Inc as the technical infrastructure provider, with audit logging of all data access events; VUNO Inc–affiliated personnel are granted no analytic or read-level access to individual-level outcome data or to aggregate treatment-allocation information before final database lock.

Access to trial data is restricted by prespecified role; site CRCs may access individual participant-level records at their respective sites, and the central PI may access the full trial dataset for trial oversight, while all other study personnel are granted access only to the data elements required to fulfill their predefined role.

At prespecified analytic time points, deidentified datasets will be exported from the trial server to a closed analytic environment maintained by the external statistical CRO via encrypted transfer. Outcome data will be analyzed by an independent statistician at the CRO, without involvement of the funders or clinicians directly providing care to enrolled participants, in accordance with the prespecified statistical analysis plan (SAP); interpretation of the analytic results will be the responsibility of the academic principal investigators, in consultation with the broader VITAL-MD study group. Research data will be retained for 3 years after completion of the trial and then securely destroyed and will not be used for secondary purposes beyond the prespecified objectives of this trial.

### Statistical Methods

#### Statistical Methods for Primary and Secondary Outcomes

The primary outcome (general ward cardiac arrest) will be analyzed using a generalized linear mixed model (GLMM) with cluster as a random effect and time and intervention as fixed effects [26]. Secondary outcomes will be analyzed using mixed effects models appropriate to outcome type. To ensure valid inference given the small number of clusters, small-sample corrections (between-within degrees of freedom) will be applied [54]. Results will be reported as adjusted risk ratios (ARR) or adjusted risk differences (ARD) with 95% CIs [55]. Models will adjust for prespecified covariates, including age, sex, department, season, weekday, NEWS2, SOFA, CCI, number of staff physicians, and bed occupancy rate.

Baseline characteristics will be compared between the intervention and the control groups using appropriate parametric or nonparametric tests for continuous variables and chi-square tests for categorical variables.

All analyses will be performed using R (version 4.3 or higher; R Foundation for Statistical Computing) or Python (version 3.7 or higher; Python Software Foundation), with the specific software and package versions reported in the primary results publication.

#### Who Will Be Included in Each Analysis

The primary analysis will be conducted in the full analysis set (FAS), consistent with the intention-to-treat (ITT) principle. The primary analysis will focus on the target cohort, defined as patients who exceed the AI-SaMD alert threshold at least once during general ward hospitalization. Patients who do not exceed the AI-SaMD alert threshold (the nontarget cohort) will be included in secondary analyses. A per-protocol set (PPS) will be used for supportive analyses. Patients admitted during the washout period and exposed to both phases (crossover) will be excluded from the primary analysis but included in sensitivity analyses.

#### How Missing Data Will Be Handled in the Analysis

The AI-SaMD system internally applies last observation carried forward (LOCF) for missing vital signs in score calculation. Missing outcome data will be reviewed by clinicians.

Missing data regarding outcomes are expected to be limited, as they are ascertained from routinely collected EMR data. The primary outcome (IHCA) and secondary outcomes subject to mandatory hospital reporting, including in-hospital mortality, LOS, and ICU transfer, are expected to have minimal missing data. The AI-SaMD internally applies LOCF to missing vital-sign inputs during real-time score computation; this is a fixed device behavior and does not affect outcome ascertainment.

#### Methods for Additional Analyses

Sensitivity analyses will include crude, propensity score matching (PSM), e-value analysis, and crossover analyses to evaluate the robustness and consistency of the primary findings [56,57]. A nontarget cohort analysis will assess the robustness of restricting the primary analysis to high-risk patients by comparing baseline characteristics, alert distribution, and patient outcomes. In addition, a full-population analysis will be conducted using the same modeling framework as the primary analysis to estimate the hospital-wide intervention effect. Time-to-event outcomes will be descriptively summarized using Kaplan-Meier estimates to visualize event timing [58]. Subgroup analyses will evaluate the consistency of the intervention effect across clinically relevant subgroups (eg, sepsis, surgical vs nonsurgical admissions, and others), including patient characteristics, clinical contexts, and reasons for or types of interventions in response to the AI-SaMD alert. A prespecified sepsis subgroup, defined by the Centers for Disease Control and Prevention (CDC) Adult Sepsis Event (ASE) surveillance criteria, will be analyzed using the same modeling framework (Multimedia Appendix 3). Lead-time analyses will evaluate changes over time in alert response time and their association with patient outcomes to assess staff response patterns and intervention adherence after implementation.

### Oversight and Monitoring

#### Trial Organization

##### Coordinating Site

The coordinating site at Inha University Hospital, which also serves as the trial sponsor, manages trial operations, including protocol implementation, data management coordination, and intersite communication. No separate external coordinating center has been established, given the relatively small number of clusters and the routinely collected data nature of this trial.

##### TMG

A TMG, composed of the principal investigators and RRS nurses at each of the 4 participating hospitals together with the trial statistician, is responsible for the day-to-day conduct of the trial, including site coordination, protocol adherence, data quality oversight, and review of operational issues. The TMG holds scheduled meetings at each cluster transition time point. Independent oversight of the trial is provided separately by the DSMB (see below) rather than through a Trial Steering Committee, given the low-risk, pragmatic nature of the trial and the use of routinely collected clinical data only.

### Data Monitoring Committee

#### DSMB

An independent DSMB, composed of independent experts in ethics, critical care, biostatistics, and clinical trials, oversees participant safety and data integrity. The DSMB reviews interim safety and efficacy data at each scheduled interim analysis. Further details are provided in the DSMB charter (Multimedia Appendix 1).

#### Interim Analyses and Stopping Guidelines

Three interim analyses will be performed at the second, third, and fourth cluster transition time points by an independent DSMB statistician. Each interim analysis will evaluate the IHCA rate (cases per 1000 admissions) after confounder adjustment and compare it with the corresponding calendar period of prior years. A >50% increase relative to this reference period will constitute a sentinel signal warranting closed-session DSMB review with clinical expert input; the DSMB will then report to the IRBs and the PI and may convene an open session to consider trial modification or termination. Further details are provided in the DSMB charter (Multimedia Appendix 1).

#### Trial Monitoring

Consistent with the risk-proportionate monitoring approach recommended by the International Council for Harmonisation (ICH) Good Clinical Practice Guideline E6(R3) and the SPIRIT 2025 statement (Item 29), and given that this is a minimal-risk pragmatic trial conducted with a waiver of individual consent and using only routinely collected EMR data, formal external on-site auditing has not been adopted. Instead, trial monitoring is conducted through a combination of (1) ongoing remote central data quality review by the CRO, including range checks, completeness checks, and consistency checks across sites; (2) periodic safety and data integrity oversight by the independent DSMB at each scheduled interim analysis; and (3) integration with the routine mortality and cardiac arrest conferences held at each of the 4 participating hospitals, as described in the Harms section, which allows independent institutional clinical judgment to flag any potential intervention-related harm directly to the site PI and the DSMB chair.

### Ethical Considerations

The study protocol was approved by the IRBs of all 4 participating hospitals: Inha University Hospital (INHAUH 2024-09-034; approved January 20, 2025), Hallym University Dongtan Sacred Heart Hospital (HDT 2024-10-006; approved March 24, 2025), Konyang University Hospital (KYUH 2024-12-004; approved December 18, 2024), and The Catholic University of Korea Incheon St. Mary’s Hospital (OC24DIDS0141; approved February 17, 2025). The requirement for informed consent was independently waived by the IRBs of all 4 participating hospitals.

Important protocol amendments will be promptly communicated to relevant parties. The principal investigator will submit revised protocols to the IRBs at all sites for approval before implementation. Approved amendments will be updated in the trial registry.

This study requests the waiving of consent from individual patients for the following three reasons: (1) this study poses minimal risk, as the AI-SaMD supplements standard care by providing nonmandatory screening recommendations without altering clinical workflows or physician autonomy; (2) the waiver will not adversely affect participants’ rights or welfare because the intervention does not restrict access to standard care; and (3) obtaining individual consent is not feasible in a large-scale hospital-based study and may introduce selection bias, as the study targets the entire inpatient population. Additional consent provisions are not applicable, as no additional participant data or biological specimens will be collected.

This study will ensure strict confidentiality throughout the data collection, analysis, and publication. All personal identifiers will be deidentified in accordance with the HIPAA requirements. Records will be retained for 3 years after completion of the trial, in accordance with the Korean Bioethics and Safety Act, and then securely disposed of. No secondary use beyond the study objectives is permitted. Ancillary and posttrial care is not applicable, as the study covers the entire general-ward hospitalization period.

## Results

The trial was registered with the Clinical Research Information Service (CRIS; KCT0010243), a World Health Organization (WHO) International Clinical Trials Registry Platform (ICTRP) primary registry in the Republic of Korea, on February 28, 2025. Of 6 tertiary academic hospitals assessed for eligibility, 4 were included; site-specific IRB approvals were obtained prior to study initiation. Patient recruitment ran from April 1, 2025, to March 31, 2026, covering the prespecified 12-month stepped-wedge schedule, and all 4 sites completed the transition to the postimplementation period. CRF finalization and data cleaning are currently underway prior to final database lock, after which statistical analysis will proceed, with the first results expected to be submitted for publication in 2027.

## Discussion

### Principal Anticipated Findings

The VITAL-MD trial will provide the first large-scale, multicenter randomized evidence on whether the deployment of an AI-EWS, used as a stand-alone clinical decision-support device—without algorithm-specific human curation or mandated workflow modification—improves patient-centered outcomes among general-ward patients in external validation environments. Building on the demonstrated diagnostic superiority of DeepCARS over conventional EWS in retrospective and prospective observational studies [19,21-24] and on the suggestive but nonrandomized signal from the recent NRCT [25], we hypothesize that the intervention period will be associated with a reduction in ward IHCA and in in-hospital mortality compared with the control group in which routine conventional EWS-based care (eg, NEWS2 and MEWS) is used. We additionally anticipate a reduction in the rate of late or missed escalations of care, an increase in proactive interventions (eg, critical care consultation, antibiotic escalation, early ICU transfer), and no clinically meaningful increase in alert burden.

### Strengths

This trial directly addresses the 2 principal limitations of the existing AI-EWS evidence base for ward-level deterioration. First, the 2 prior multicenter cluster RCTs for ward deterioration—the negative CoMET trial and the positive CONCERN trial, currently representing the highest-level randomized evidence in this field—were both conducted within their internal development environments [14,15]. Because AI model performance frequently drops substantially when transferred to external settings due to dataset shifts [59], whether these divergent outcomes generalize beyond their developing institutions remains unestablished. To our knowledge, VITAL-MD will be the first multicenter randomized trial to evaluate the patient outcome effect of an AI-EWS in true external validation environments. Second, previous interventional studies bundled algorithms with specific implementation infrastructures, such as off-site nurse curation in the Advance Alert Monitor deployment [28]. By deploying DeepCARS as a stand-alone device without algorithm-specific human curation or mandated workflow modification, VITAL-MD isolates the pure effect of the AI-SaMD itself from the surrounding human and workflow scaffolding. Furthermore, establishing causal outcome benefits for rapid response mechanisms has historically been challenging. Comprehensive Cochrane systematic reviews and the landmark Medical Early Response, Intervention, and Therapy (MERIT) trial demonstrated little or no significant reduction in unexpected mortality from conventional RRS and EWS interventions [8,60]. Despite this, major societies such as the AHA and SCCM continuously and strongly recommend hospital-wide surveillance systems [4,5]. VITAL-MD aims to bridge this critical evidence gap by generating definitive causal evidence that replacing the inaccurate afferent limb with a highly accurate AI-EWS can validate the clinical rationale behind these ongoing recommendations. Evaluating the intervention directly against usual clinical practice and the standard of care is strongly emphasized by major guidelines as the most rigorous benchmark for establishing clinical utility [11]. The SW-CRT design robustly controls for secular trends and between-cluster confounding, evaluating this real-world standard across a massive cohort of more than 70,000 anticipated admissions.

### Shifting From Explainability to Actionability

Furthermore, this study seeks to propose a novel paradigm for understanding and evaluating AI outputs, pivoting the focus from algorithmic “explainability” to clinical “actionability.” The prevailing approach to mitigating the “black-box” nature of deep learning models involves post hoc mathematical interpretability techniques, such as Shapley Additive Explanations (SHAP) values. However, emerging literature clearly indicates that such mathematical explanations fail to translate into tangible clinical actions. As highlighted by Bienefeld et al [61], mathematical feature weightings do not provide clinical actionability and are frequently perceived by clinicians as unhelpful in acute care settings. We will therefore investigate the specific clinical mechanisms driving outcome improvements by prospectively analyzing lead times to targeted clinical interventions (eg, critical care consultation, antibiotic escalation, and early ICU consultation) and identifying which clinical subgroups benefit most from actions prompted by AI alerts. Providing HCPs with practical, action-oriented guidelines on how to respond to AI alerts will directly maximize the real-world utility of the AI-SaMD.

### Limitations

Several limitations warrant explicit acknowledgment. First, while this study provides causal evidence for one specific algorithm, clinical efficacy is not a universal characteristic of all AI-EWS platforms. Emerging literature demonstrates that commercial medical AI devices exhibit vast disparities in their real-world performance [16]. Recent multicenter evaluations have shown that sensitivities, positive predictive values, and false-alarm burdens range widely across AI products even when evaluating the exact same clinical deterioration targets [9,62,63]. Consequently, the patient-centered benefits observed in this trial apply exclusively to the implemented AI-SaMD and do not validate the universal clinical utility of commercial early warning algorithms. Second, while all general ward admissions are included in the analysis, the primary evaluation focuses predominantly on the “target cohort,” patients who exceed the high-risk alert threshold. Although restricting the primary analysis to this subset limits inferences about the overall unselected hospital population, this targeted methodology perfectly aligns with the algorithm’s intended clinical use as a medical device to identify and trigger responses for high-risk patients. Furthermore, this approach is consistent with prior AI-EWS evaluations, such as the studies by Escobar et al [28] and Winslow et al [29], justifying its analytical validity for evaluating algorithmic rapid response interventions. Third, the study is conducted exclusively in tertiary academic hospitals within South Korea. The lack of racial and geographic diversity limits the direct generalizability of the findings to health care systems in other countries or nontertiary settings. Fourth, the planned number of randomized clusters was scaled down to 4 sites, which not only decreases statistical efficiency but also means the between-cluster variance is imprecisely estimated; we address the resulting inferential limitations through the stepped-wedge structure and prespecified small-sample corrections [26,54]. Finally, the passive, nonmandatory nature of the AI intervention might lead to variable compliance and alert fatigue among clinicians over time. Potential cross-contamination during transition periods will be carefully monitored via strict washout period definitions and prespecified sensitivity analyses.

### Implications for Research, Practice, and Policy

If the trial demonstrates a beneficial effect of DeepCARS on patient-centered outcomes, it will set a methodological benchmark for AI-EWS evaluation, complementing prior multicenter cluster RCTs by being the first conducted in external validation environments as a stand-alone device. For clinical practice, it will provide direct evidence on whether an AI-EWS can improve outcomes in resource-limited or workforce-constrained settings where additional curating personnel are not feasible. For regulatory science, it will inform the ongoing debate on whether AI-SaMDs—currently approved primarily based on retrospective diagnostic performance—should also be required to demonstrate prospective effects on patient-centered outcomes before or shortly after market authorization [13].

### Conclusions

The VITAL-MD trial is designed to generate the first multicenter randomized evidence on whether an AI-EWS, deployed as stand-alone software as a medical device in external validation environments, can improve patient-centered outcomes for general-ward patients. By directly comparing the intervention with usual care and prospectively characterizing the clinical actions that mediate any observed benefit, the trial seeks to advance AI-SaMD evaluation from retrospective diagnostic accuracy toward prospective, action-oriented clinical utility, with anticipated implications for clinical practice, hospital policy, and regulatory science.

### Dissemination Policy

#### Dissemination of Trial Results

Trial results will be disseminated regardless of the direction or statistical significance of the findings. The primary results manuscript will be submitted to a peer-reviewed international journal within 12 months after final database lock and prepared in accordance with the CONSORT (Consolidated Standards of Reporting Trials) 2025 statement. Findings will be presented at relevant scientific conferences in critical care medicine, RRS, patient safety, cardiovascular disease, and biomedical informatics. Authorship of the primary results manuscript will follow the International Committee of Medical Journal Editors (ICMJE) criteria. The author list will be finalized before database lock, based on contributions to trial conception, design, conduct, analysis, or interpretation. Members of the VITAL-MD Study Group who do not meet ICMJE authorship criteria will be acknowledged as collaborators.

The TMG retains sole authority over the review and approval of all trial-related publications and presentations. The executive leadership of Inha University Hospital and all other funding organizations are strictly prohibited from editing, approving, or delaying the publication of the trial outcomes.

#### Access to the SAP, Participant-Level Data, and Statistical Code

The SAP is provided as Multimedia Appendix 4. Deidentified data and analysis code will be made available from March 2027 upon reasonable request to JSK (Inha University Hospital), subject to ethical approval. The dataset will include each participant’s vital signs, AI-SaMD scores, intervention type and reason, and corresponding clinical outcomes.

## Supplementary material

10.2196/104230 Multimedia Appendix 1 Data and Safety Monitoring Board (DSMB) charter.

10.2196/104230 Multimedia Appendix 2 Randomization procedure and intervention time point allocation in a stepped-wedge cluster randomized trial (SW-CRT) for the 4 participating sites.

10.2196/104230 Multimedia Appendix 3 Supplementary methods.

10.2196/104230 Multimedia Appendix 4 Statistical analysis plan (SAP).

10.2196/104230 Checklist 1 SPIRIT 2025 checklist.

## Abbreviations

AHA: American Heart Association
AI-EWS: AI-based Early Warning Score
AI-SaMD: AI-based software as a medical device
ARD: adjusted risk difference
ARR: adjusted risk ratio
ASE: Adult Sepsis Event
AUROC: area under the receiver operating characteristic curve
CCI: Charlson Comorbidity Index
CDC: Centers for Disease Control and Prevention
CoMET: Continuous Monitoring of Event Trajectories
CONCERN: Communicating Narrative Concerns Entered by Registered Nurses
CONSORT: Consolidated Standards of Reporting Trials
CPC: Cerebral Performance Category
CPR: cardiopulmonary resuscitation
CRC: clinical research coordinator
CRF: case report form
CRIS: Clinical Research Information Service
CRO: contract research organization
DECIDE-AI: Developmental and Exploratory Clinical Investigations of Decision Support systems driven by AI
DNR: do-not-resuscitate
DSMB: Data and Safety Monitoring Board
EMR: electronic medical record
EWS: early warning score
FAS: full analysis set
GLMM: generalized linear mixed model
HCP: health care professional
HIPAA: Health Insurance Portability and Accountability Act
ICC: intracluster correlation coefficient
ICH: International Council for Harmonisation
ICMJE: International Committee of Medical Journal Editors
ICTRP: International Clinical Trials Registry Platform
ICU: intensive care unit
IHCA: in-hospital cardiac arrest
IRB: institutional review board
ITT: intention-to-treat
*JAMA*: *Journal of the American Medical Association*
LOCF: last observation carried forward
LOS: length of stay
MERIT: Medical Early Response, Intervention and Therapy
MEWS: Modified Early Warning Score
ML: machine learning
NEWS2: National Early Warning Score 2
NRCT: nonrandomized controlled trial
PI: principal investigator
PPS: per-protocol set
PSM: propensity score matching
RCT: randomized controlled trial
RRR: relative risk reduction
RRS: rapid response system
SAP: statistical analysis plan
SCCM: Society of Critical Care Medicine
SHAP: Shapley Additive Explanations
SOFA: Sequential Organ Failure Assessment
SPIRIT: Standard Protocol Items: Recommendations for Interventional Trials
SpO_2_: oxygen saturation
SW-CRT: stepped-wedge cluster randomized trial
TMG: trial management group
UIT: unplanned intensive care unit transfer
VITAL-MD: validation of an AI-based clinical deterioration prediction model on patient outcomes through a stepped-wedge cluster randomized trial: multicenter evaluation of DeepCARS
WHO: World Health Organization
