# Supplementary material for: Clinical Effectiveness of AI-Based Cardiac Arrest Prediction System for General Ward Patients (VITAL-MD): Protocol for a Multicenter Pragmatic Stepped Wedge Cluster Randomized Controlled Trial
Source: JMIR Res Protoc. 2026 Oct 19;15:e104230. doi: 10.2196/104230 (PMC13679675; doi:10.2196/104230)
Supplement: Supplementary file 1 [file resprot-v15-e104230-s002.pdf]

Randomization Procedure and Intervention Time Point Allocation in a Stepped-Wedge Cluster Randomized Trial (SW-CRT) for Four Sites

This document outlines the randomization procedure used in a Stepped-Wedge Cluster Randomized Trial (SW-CRT) involving four participating sites. The trial spans one year and includes 10 defined time points.

The purpose of this procedure is to assign each of the four sites randomly to one of the predefined intervention initiation time points: **2.4, 4.8, 7.2, and 9.6** (representing cumulative time in months). These assignments are designed to ensure unbiased implementation timing across sites while preserving the integrity of the randomization process.

All randomization procedures were conducted under strict controls to maintain allocation concealment and prevent selection bias. The final assignment results and related metadata were saved in **RTF and plain text formats** to ensure traceability and reproducibility.

1 Overview

This document describes the randomization procedure used to assign intervention initiation time points to four hospitals (A, B, C, and D) within the framework of a Stepped-Wedge Cluster Randomized Trial (SW-CRT) design. The study spans 10 time points over one year, with fixed intervention start points set at 2.4, 4.8, 7.2, and 9.6 months.

The purpose of this randomization is to ensure the validity of the trial by preventing allocation bias and enhancing the reliability of the estimated intervention effect. The randomization is implemented in two steps:

- In **Step 1**, a random number is assigned to each hospital (A to D).
- In **Step 2**, the hospitals are randomly reordered based on these numbers and assigned to one of the four fixed intervention time points.

This two-stage procedure guarantees proper allocation concealment and ensures the scientific rigor of the intervention rollout. All procedures are timestamped, and the output files are stored in **RTF and plain text formats** for traceability and data integrity.

This documentation serves as formal evidence of the randomization logic and contributes to the transparency of the trial design and execution.

2 Key Features

- **Random assignment of hospitals:** Four hospitals are randomly allocated to one of four predefined intervention time points.
- **Timestamping:** The date and time of random number generation are recorded.
- **File storage:** The final assignment results are saved in both RTF and text formats for recordkeeping.

3 Variable Definitions

Variable	Description
hospital	The name of the participating hospital (Hospital A, B, C, D)
site	The randomly assigned number for each hospital
intervention_time	The fixed intervention initiation time point (2.4, 4.8, 7.2, or 9.6 months)
creation_datetime	The timestamp of random assignment, generated using the datetime() function

4 SAS Code: Randomization Steps

Step 1: Assign Random Numbers to Hospitals
Explanation:

- data hospitals defines the list of participating hospitals (병원 A, 병원 B, 병원 C, 병원 D).

- proc plan randomly assigns a site number (1–4) to each hospital using a fixed seed (12345) for reproducibility.
- A timestamp of the assignment is recorded using datetime().
- The final output dataset rand_hospitals includes the hospital name, random site number, and generation timestamp.

```
/* Step 1: Define hospital names */
data hospitals;
  input hospital $;
  datalines;
병원 A
병원 B
병원 C
병원 D
;
run;

/* Assign random numbers to the hospitals using PROC PLAN */
proc plan seed=12345;
  factors site=4 random;
  output out=rand_hospitals;
run;

/* Merge hospital names with random numbers and add timestamp */
data rand_hospitals;
  merge hospitals rand_hospitals;
  retain hospital site;
  creation_datetime = datetime(); /* Timestamp of randomization */
  format creation_datetime datetime20.;
run;

proc sort data=rand_hospitals; by site; run;
   ods rtf close;
%mend;
```

Step 2: Assign Randomized Intervention Time Points

Based on the random numbers assigned in Step 1, each hospital is allocated to one of the predefined intervention initiation time points: **2.4, 4.8, 7.2, or 9.6 months**. The order of allocation is randomized to ensure unbiased distribution of intervention timing across the four participating hospitals.

```
/* Step 2: Randomly shuffle intervention time points */
proc plan seed=67891;
  factors time=4 random;
  output out=rand_times;
run;
```

```
/* Merge hospital data with randomized time point assignment */
data final_hospitals;
  merge rand_hospitals rand_times;
         if time = 1 then intervention_time = 2.4;
  else if time = 2 then intervention_time = 4.8;
  else if time = 3 then intervention_time = 7.2;
  else if time = 4 then intervention_time = 9.6;
run;

proc sort data=final_hospitals; by time; run;
```

Final Output and File Export

The final randomization result, including the assigned intervention time point for each hospital, is saved in both **RTF** and **text** file formats for documentation and audit purposes.

```
/* RTF Output*/
ods rtf file=' [Path]/intervention_randomization_results.rtf';
proc print data=final_hospitals noobs;
  var hospital site intervention_time creation_datetime;
run;
ods rtf close;

/* Text File Output*/
proc export data=final_hospitals
  outfile=' [Path]/intervention_randomization_results.txt’
  dbms=dlm
  replace;
  delimiter=' ';
run;
```

5 Seed Value Configuration

The seed value used in randomization controls the starting point for pseudo-random number generation. Using fixed seed values ensures that the randomization procedure is reproducible, which is essential for transparency and auditability in clinical research.

In the provided code, seed=12345 and seed=67891 are used to generate consistent and reproducible random assignments.

Example Seed Configuration:

1. Random number assignment to hospitals:

```
    proc plan seed=12345;
      factors site=4 random;  /* Assign random numbers to 4 hospitals */
      output out=rand_hospitals;
    run;
```

2. Randomized intervention time point allocation:

```
proc plan seed=67891;
  factors time=4 random;  /* Random allocation of fixed time points*/
  output out=rand_times;
run;
```

If necessary, different seed values may be used to enhance randomness or to generate a new randomization sequence.

6 Post-Execution Output Files

- Excel-compatible text file: The final result is exported as a .txt file using space as the delimiter. This file can be opened and reviewed in Excel.
- Formatted RTF file: The result is also saved as a .rtf document for formal reporting and archiving.

7 Final Randomization Result

The final randomization assigns each hospital a random number and intervention start time. An example output might be as follows:

2024년 9월 30일 월요일 오전 09시 53분

SAS 시스템

hospital	site	intervention_time	creation_datetime
병원A	2	2.4	30SEP2024:11:06:45
병원C	1	4.8	30SEP2024:11:06:45
병원D	3	7.2	30SEP2024:11:06:45
병원B	4	9.6	30SEP2024:11:06:45

파일 편집 보기

```
hospital site creation_datetime time intervention_time
병원A 2 30SEP2024:11:06:45 1 2.4
병원C 1 30SEP2024:11:06:45 2 4.8
병원D 3 30SEP2024:11:06:45 3 7.2
병원B 4 30SEP2024:11:06:45 4 9.6
```

기관별 무작위 배정 결과

2025.3.26, 씨앤케이인사이트

1. 연구 개요

본 연구는 DeepCARS 시스템이 병원 내 심정지 발생률에 미치는 효과를 평가하고자 한다. 심정지 발생률 감소 효과를 입증하기 위해 Stepped Wedge 연구 디자인을 적용하였으며, 처치군과 대조군 간의 심정지 발생률 차이를 통계적으로 분석하여 그 효과를 평가한다.

2. 무작위 배정 절차

무작위 배정은 다음 단계에 따라 진행되었습니다.

1. 병원 목록 작성

   본 연구에 참여하는 병원은 다음 4개 기관입니다:

   - 건양대병원
   - 동탄성심병원
   - 인천성모병원
   - 인하대병원

2. 이름순 정렬 및 병원 코드 할당

   병원명을 이름순으로 정렬하여 다음과 같이 병원 코드(A, B, C, D)를 부여하였습니다:

   - A. 건양대병원
   - B. 동탄성심병원
   - C. 인천성모병원
   - D. 인하대병원

3. Seed 값 자동 생성

   본 무작위 배정을 위하여 기관 및 중재 시점에 대해 무작위 배정을 실시하도록 계획되었습니다. 무작위 배정을 위한 Seed 값은 프로그램이 자동으로 생성하도록 설계하여 연구자가 Seed 값을 임의로 지정하는 개입을 방지하였습니다.

   Seed 값 자동 생성을 통해 (59908, 72812)가 생성되었습니다.

   본 보고서에 명시된 Seed 값 (59908, 72812)은 이미 확정된 값이므로 해당 값을 변경하지 않도록 주의해 주시기 바랍니다.

   주의) 해당 프로그램을 다시 실행할 경우 Seed 값이 변경되며 결과가 달라질 수 있습니다.

4. 기관 무작위 배정

   PROC PLAN을 이용하여 4개 기관을 대상으로 무작위 배정을 수행하였습니다.

   이 과정에서 Seed 값으로 59908을 설정하여, 향후 동일한 결과를 재현할 수 있도록 하였습니다.

5. 중재 시점 무작위 배정

PROC PLAN을 이용하여 각 기관의 중재 시점을 무작위로 배정하였습니다.
이 과정에서 Seed 값으로 72812을 설정하여 동일한 배정 결과를 재현할 수 있도록 하였습니다.

6. 최종 배정 결과 생성
   위의 두 단계에서 생성된 무작위 번호에 따라 각 기관의 중재 시점을 확정하고, 해당 정보를 표로 정리하였습니다.

3. SEED 값 및 역할

1. **Seed 1 (기관 무작위 배정)**: 59908
2. **Seed 2 (중재 시점 무작위 배정)**: 72812
3. 해당 SEED 값은 향후 재현 가능성을 확보하기 위한 고유 식별 값입니다. 연구의 공정성과 신뢰성을 확보하기 위해 사용된 중요한 요소입니다.

4. 무작위 배정 결과

Generated Seed Values

seed1	seed2
59908	72812

Generated Seed Values

The PLAN Procedure

Factor	Select	Levels	Order
site	4	4	Random

site			
3	2	4	1

Generated Seed Values

hospital	site	creation_datetime
D.인하대병원	1	26MAR2025:08:44:52
B.동탄성심병원	2	26MAR2025:08:44:52
A.건양대병원	3	26MAR2025:08:44:52
C.인천성모병원	4	26MAR2025:08:44:52

Generated Seed Values

The PLAN Procedure

Factor	Select	Levels	Order
time	4	4	Random

time			
2	1	3	4

Generated Seed Values

hospital	site	intervention_time	intervention_date	creation_datetime
B.동탄성심병원	2	Post-1st	13JUN2025	26MAR2025:08:44:52
D.인하대병원	1	Post-2nd	27AUG2025	26MAR2025:08:44:52
A.건양대병원	3	Post-3rd	10NOV2025	26MAR2025:08:44:52
C.인천성모병원	4	Post-4th	23JAN2026	26MAR2025:08:44:52

5. 무작위 배정 파일 자동 결과

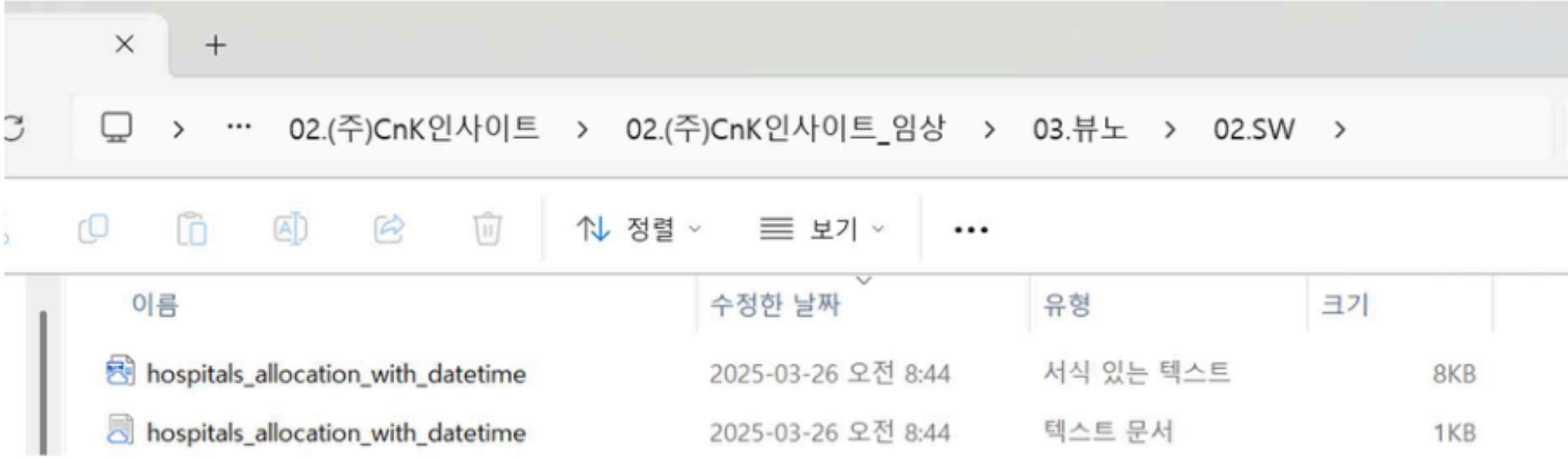

6. 문의

- 문의 사항이 있을 경우 아래의 연락처로 문의해 주시기 바랍니다.
- [redacted]

Appendix. SAS 프로그램 코드

```
/* Step 0: 병원 A, B, C, D 생성 */
data hospitals;
   length hospital $20;
   input hospital $;
   datalines;
A.건양대병원
B.동탄성심병원
C.인천성모병원
D.인하대병원
;
run;

/* Step 1: 기관 및 중재 시점을 위한 Seed 값 생성 */
data seeds;
   seed1 = floor(ranuni(0) * 100000); /* 0~99999 사이의 랜덤한 정수 */
   seed2 = floor(ranuni(0) * 100000); /* 0~99999 사이의 랜덤한 정수 */
run;

proc print data=seeds noobs;
   title 'Generated Seed Values';
run;

/* Step 2: 기관 무작위 배정 */
data _null_;
   set seeds;
```

```
   call symputx('seed1', seed1);
run;

proc plan seed=&seed1;
   factors site=4 random;
   output out=rand_hospitals;
run;

/* 병원과 무작위 번호 병합, 번호 생성일시 추가 */
data rand_hospitals;
   merge hospitals rand_hospitals;
   retain hospital site;
   creation_datetime = datetime();
   format creation_datetime datetime20.;
run;

proc sort data=rand_hospitals; by site; run;

proc print data=rand_hospitals noobs;
run;

/* Step 3: 중재 시점 무작위 배정 */
data _null_;
   set seeds;
   call symputx('seed2', seed2);
run;
```

```
proc plan seed=&seed2;
   factors time=4 random;
   output out=rand_times;
run;

/* Step 4: 병원에 부여된 번호에 따른 중재 시점 매핑 */
data final_hospitals;
   merge rand_hospitals rand_times;

   if time = 1 then intervention_date = '13JUN2025'd;
   else if time = 2 then intervention_date = '27AUG2025'd;
   else if time = 3 then intervention_date = '10NOV2025'd;
   else if time = 4 then intervention_date = '23JAN2026'd;

   format intervention_date date9.;

   if time = 1 then intervention_time = 'Post-1st';
   else if time = 2 then intervention_time = 'Post-2nd';
   else if time = 3 then intervention_time = 'Post-3rd';
   else if time = 4 then intervention_time = 'Post-4th';
run;

proc sort data=final_hospitals; by intervention_date; run;

/* Step 5: 최종 결과 출력 */
```

```
proc print data=final_hospitals noobs;
   var hospital site intervention_time intervention_date creation_datetime;
run;



/* 데이터 텍스트 파일로 내보내기 (생성일시 포함) */
proc export data=final_hospitals
   outfile='G:₩내 드라이브₩AA.Chung&Kang₩02.(주)CnK인사이트₩02.(주)CnK인사이트_임상₩03.뷰노₩02.SW/hospitals_allocation_with_datetime.txt'
   dbms=dlm
   replace;
   delimiter=' ';
run;

/* ODS RTF로 데이터 출력 (Word에서 열 수 있는 형식) */
ods rtf file='G:₩내 드라이브₩AA.Chung&Kang₩02.(주)CnK인사이트₩02.(주)CnK인사이트_임상₩03.뷰노₩02.SW/hospitals_allocation_with_datetime.rtf';
proc print data=final_hospitals noobs;
   var hospital site intervention_time intervention_date creation_datetime;  /* 열 순서 지정, 생성일시 포함 */
run;
ods rtf close;
```
