# Supplementary material for: Clinical Effectiveness of AI-Based Cardiac Arrest Prediction System for General Ward Patients (VITAL-MD): Protocol for a Multicenter Pragmatic Stepped Wedge Cluster Randomized Controlled Trial
Source: JMIR Res Protoc. 2026 Oct 19;15:e104230. doi: 10.2196/104230 (PMC13679675; doi:10.2196/104230)
Supplement: Supplementary file 2 [file resprot-v15-e104230-s001.docx]

**Data and Safety Monitoring Board (DSMB) Charter**

**Clinical effectiveness of artificial intelligence-based prediction model for cardiac arrest in general ward-admitted patients (VITAL-MD): a structured study protocol for multicenter pragmatic cluster randomized trial**

**Date**

2025-01-20

**Protocol version**

INHAUH 2024-09-034 (version 001)

**Principal Investigator**

Jungsoo Kim, MD, MS

Department of Critical Care Medicine,

Inha University, Incheon, Republic of Korea

**Sponsor**

Inha University Hospital

27, Inhang-ro, Jung-gu, Incheon, Republic of Korea

The Data and Safety Monitoring Board (DSMB) will act in an advisory capacity to the Institutional Review Boards (IRBs) and the Principal Investigator to monitor participant safety, data quality and evaluate the progress of the study.

**DSMB Responsibilities**

The DSMB responsibilities are to:

- review the research protocol and plans for data safety and monitoring;
- advise the IRBs and the Principal Investigator on the readiness of the participating sites to initiate AI-SaMD implementation;
- evaluate the progress of the trial, including periodic assessments of data quality and timeliness, case ascertainment and data completeness, participant risk versus benefit, performance of the trial sites, and other factors that can affect study outcome;
- consider factors external to the study when relevant information becomes available, such as scientific or therapeutic developments that may have an impact on the safety of the participants or the ethics of the trial;
- review study performance, make recommendations and assist in the resolution of problems reported by the Principal Investigator;
- protect the safety of the study participants;
- report to the IRBs and the Principal Investigator on the safety and progress of the trial;
- make recommendations to the IRBs and the Principal Investigator concerning continuation, termination or other modifications of the trial based on the observed beneficial or adverse effects of the intervention under study;
- if appropriate, review interim analyses in accordance with stopping rules, which are clearly defined in advance of data analysis and have the approval of the DSMB;
- ensure the confidentiality of the study data and the results of monitoring; and,
- assist the Trial Management Group (TMG) by commenting on any problems with study conduct, data completeness, sample size, and/or data collection.

The DSMB will discharge itself from its duties when the last participant completes the study.

**Membership**

Most often DSMB consists of five members and three members usually constitute a quorum. However, the number of DSMB members varies depending on the size and complexity of the trial and may vary from three to nine or more members. When the DSMB consists of more than five individuals, at least 50% of members must be present at the meeting to constitute the quorum. Membership consists of individuals who have no financial, scientific, or other conflict of interest with the trial. Collaborators or associates of the investigator are not eligible to serve on the DSMB. Written documentation attesting to absence of conflict of interest is required.

The DSMB includes experts in or representatives of the fields of:

- Ethicists
  - Hyuna Bae, MD, PhD (Chairperson)
  - Ewha Womans University School of Law
- Statistician
  - Jaehun Jung, MD, PhD
  - Department of Preventive Medicine, Korea University College of Medicine
- Clinical expertise
  - Tai Sun Park, MD, PhD
  - Division of Pulmonary Medicine and Allergy, Department of Internal Medicine, Hanyang University College of Medicine
- Clinical trial methodology
  - Sooyeon Chung, MS
  - CEO, C&K INSIGHT Co., Ltd.

The Chairperson is responsible for overseeing the meetings and developing the agenda in consultation with the IRBs and the Principal Investigator. The Chair is the contact person for the DSMB. The sponsor (Inha University Hospital) shall provide the logistical management and support of the DSMB.

A safety officer maybe identified at the first meeting and is typically the Chair unless this individual is not a physician. In this case another member of the DSMB is appointed as the safety officer. The safety officer will be the contact person for serious adverse event reporting. Procedures for notifying the Chair of the DSMB will be discussed and agreed upon at the first meeting.

**Board Process**

At the first meeting the DSMB will discuss the protocol, suggest modifications, and establish guidelines to study monitoring by the Board. The DSMB Chairperson in consultation with the Principal Investigator will prepare the agenda to address the review of study materials, modifications to the study protocol, initiation of the trial, appointment of a safety officer, as needed, reporting of adverse events, statistical analysis plan including interim analysis and stopping rules, etc.

Data safety monitoring will be conducted during three of the four transition periods from the stepped wedge cluster randomized trial (SW-CRT) pre-implementation phase to the post-implementation phase, excluding the first transition. Interim analyses will be performed in accordance with these monitoring periods, and DSMB meetings will be held to review the results. An emergency meeting of the DSMB may be called at any time by the Chair or by the Principal Investigator or the Trial Management Group if participant safety concerns or other unanticipated problems arise.

Meetings are closed to the public because discussions may address confidential participant data. Meetings are attended by the Principal Investigator and members of his/her staff. Meetings may be convened as conference calls as well as in-person.

**Communications**

To avoid any appearance of conflict of interest, routine communications between the investigators and the DSMB on analytic and interim-data matters should not be conducted directly but should be channelled through the coordinating site (Inha University Hospital). This includes protocols, the manual of procedures, interim reports, recommendations, and other routine study-related correspondence.

Notwithstanding the above, any concern about potential intervention-related harm—including concerns raised at the routine mortality and cardiac arrest conferences at the participating hospitals—may be reported directly to the site Principal Investigator and the DSMB Chair, who serves as the DSMB contact person, at any time by telephone and email. All handling of study data otherwise follows the Health Insurance Portability and Accountability Act (HIPAA) de-identification standard as described elsewhere in this document.

**Meeting Format**

DSMB meetings will consist of open and closed sessions. Discussion held in all sessions is confidential. The Principal Investigator and key members of the study team attend the **open sessions.** Open session discussion will focus on the conduct and progress of the study, including case ascertainment, protocol compliance, and problems encountered. Unblinded data are not presented in the open session.

The **closed session** will be attended by the DSMB members. The study statistician may be present, at the request of the DSMB. Any data by blinded study group and, as necessary, unblinded data, are presented during the closed session.

If necessary, an **executive session** will be attended by voting DSMB members and the Principal Investigator (or a designated Trial Management Group representative). The executive session will be held to identify and discuss the DSMB’s recommendations. The study staff may be present, at the request of the DSMB, during the executive session.

Each meeting must include a recommendation to continue or to terminate the study and whether the DSMB has any concerns about participant safety made by a formal DSMB majority or unanimous vote. Should the DSMB decide to issue a termination recommendation, the full vote of the DSMB is required. In the event of a split vote, majority vote will rule and a minority report should be appended. The DSMB Chair provides the tiebreaking vote in the event of a 50-50 split vote.

A recommendation to terminate the study may be made by the DSMB at any time by majority vote. The Chair should provide such a recommendation to the IRBs immediately by telephone and email. After the IRBs Director makes a decision about whether to accept or decline the DSMB recommendation to terminate the study, the Principal Investigator is immediately informed about his decision.

**Meeting Materials**

DSMB interim report templates will be prepared by the study staff, typically the statistician, to be reviewed by the DSMB members at the first meeting. Interim data reports generally consist of two parts:

- Part 1 - Open Session Report and
- Part 2 - Closed Session Report

Format and content of the reports for both the open and closed sessions and plans for interim analyses should be finalized and approved at the initial DSMB meeting, although changes throughout the trial may be requested by the Board.

The reports will list and summarize safety data and describe the status of the study. The reports are numbered and provided in sealed envelopes within an express mailing package or by secure email as the DSMB prefers.

- **Part 1 - Open Session Reports:** Open session reports generally include administrative reports by site that describe patients admitted and monitored, with outcomes ascertained, as well as baseline characteristics of the study population. Other general information on study status may also be presented. Listings of adverse events and serious adverse events as well as any other information requested by the DSMB may also be in the open session report, but none of the data should be presented in an unblinded manner. The DSMB may direct additions and other modifications to the reports on a one-time or continuing basis.
- **Part 2 – Closed Session Report:**  Closed session reports generally present the same information as presented in the open session but by blinded study group (e.g. A/B, etc.). The reports may also contain data on study outcomes, including safety data, and depending on the study, perhaps efficacy data. The Closed Session reports should be destroyed at the conclusion of the meeting. If the meetings are held by telephone,

**Additional Reports**

- **Mailings to the DSMB:** On a scheduled basis (as agreed upon by the DSMB) blinded safety data could be communicated to all DSMB members or to the designated Safety Officer. The study may provide these data directly to the DSMB, if such communication procedures are approved by the IRBs.
- **Access to Interim Data:** Access to the accumulating endpoint data should be limited to as small a group as possible, such as the statistician. Limiting the access to interim data to the DSMB members relieves the investigators of the burden of judging whether it is ethical to continue the trial and helps protect the study from bias in trial conduct and/or evaluation.
- **Interim Analyses:** In studies in which an interim analysis is planned, the DSMB will need to review investigators’ plan and advise the IRBs on its appropriateness (e.g., at prespecified cluster transition timepoints). The interim analysis will be reviewed in closed session and may be presented in blinded group A/B format; however the DSMB may request review of the unblinded data.

Interim analyses are performed according to the prespecified monitoring schedule, with criteria for possible modification or termination clearly defined in advance. Three interim analyses will be performed at the second, third, and fourth cluster transition timepoints by an independent DSMB statistician. Each interim analysis will evaluate the in-hospital cardiac arrest (IHCA) rate, defined as the number of IHCA cases per 1,000 admissions, after adjustment for confounders, and compare it with the corresponding calendar period of prior years. A greater than 50% increase in the adjusted IHCA rate relative to this reference period will constitute a sentinel safety signal warranting closed-session DSMB review with clinical expert input; the DSMB will then report to the IRBs and the Principal Investigator and may convene an open session to consider modification or termination of the trial.

**Reports from the DSMB**

A formal report containing the recommendations for continuation or modifications of the study will be prepared by the DSMB Chairperson. The draft report will be sent to the DSMB members for review and approval. Once approved by the DSMB members, the Chairperson will forward the formal DSMB recommendation to the Principal Investigator. It is the responsibility of the Principal Investigator to distribute the DSMB recommendation to all co-investigators and to ensure that copies are submitted to all the IRBs associated with the study.

As previously stated, the formal DSMB report must include a recommendation to continue or to terminate the study. This recommendation should be made by formal majority vote. A termination recommendation may be made by the DSMB at any time by majority vote. The IRBs are responsible for notifying the Principal Investigator of its decision to terminate the study. In the event of a split vote in favor of continuation, a minority report should be contained within the regular DSMB report. The report should not include unblinded data or discussion of the unblinded data.

**Confidentiality**

All materials, discussions and proceedings of the DSMB are completely confidential. Members and other participants in DSMB meetings are expected to maintain confidentiality.
