# Supplementary material for: Clinical Effectiveness of AI-Based Cardiac Arrest Prediction System for General Ward Patients (VITAL-MD): Protocol for a Multicenter Pragmatic Stepped Wedge Cluster Randomized Controlled Trial
Source: JMIR Res Protoc. 2026 Oct 19;15:e104230. doi: 10.2196/104230 (PMC13679675; doi:10.2196/104230)
Supplement: Supplementary file 3 [file resprot-v15-e104230-s003.docx]

**Supplementary Methods**

This appendix provides the full operational definitions of (1) the admission Sequential Organ Failure Assessment (SOFA) score adapted for general-ward EMR data availability and (2) the prespecified sepsis subgroup based on the Centers for Disease Control and Prevention (CDC) Adult Sepsis Event (ASE) surveillance framework.

Contents:

- Supplementary Methods 1. Detailed description of SOFA score at admission
- Supplementary Methods 2. Operational definition of sepsis based on ASE criteria

**Supplementary Methods 1. Detailed description of SOFA score at admission**

The Sequential Organ Failure Assessment (SOFA) score, originally developed to quantify the degree of organ dysfunction in critically ill patients across six organ systems (respiratory, cardiovascular, hepatic, coagulation, renal, and neurological), has since been endorsed by the Third International Consensus Definitions for Sepsis and Septic Shock (Sepsis-3) [Singer et al., 2016] as the reference operationalization of sepsis-defining organ dysfunction, in which an acute rise of ≥2 points denotes life-threatening organ failure (Box 1). In this trial, illness severity at the time of admission will be quantified by the admission SOFA score, computed from the first available measurement of each component during the index hospitalization.

A direct application of the original SOFA construct, however, is not feasible on general wards. Several of its canonical inputs—the PaO₂/FiO₂ ratio, mean arterial pressure (MAP), the Glasgow Coma Scale (GCS), and in particular, the dose-resolved vasopressor information required for cardiovascular grading—are seldom captured in non-ICU electronic records with the granularity that the original SOFA scoring assumes. To preserve the conceptual content of SOFA while remaining computable from ward-level data, baseline severity in this trial will be operationalized through a ward-adapted SOFA formulation aligned with the framework proposed by Raymond et al. [Raymond et al.], with study-specific refinements detailed below.

**Operationalization and Scoring**

**Cardiovascular and respiratory components.** The two subscores that depend most heavily on ICU-specific inputs are re-defined using measurements that are routinely available on general wards. The cardiovascular subscore is based on systolic blood pressure (SBP), graded according to whether intravenous fluids or inotropes have been administered, in place of mean arterial pressure (MAP) combined with detailed vasopressor dosing. The respiratory subscore is based on peripheral oxygen saturation (SpO₂), graded according to whether supplemental oxygen or mechanical ventilation is in use, in place of the PaO₂/FiO₂ ratio.

**Central nervous system component.** The CNS subscore is operationalized using the AVPU (Alert / Verbal / Pain / Unresponsive) scale, which is documented on general wards far more consistently than GCS. A score of 0 is assigned to an "Alert" patient (AVPU A, equivalent to GCS 15), and a score of 1 is assigned to any other state (AVPU V, P, or U).

**Hepatic, coagulation, and renal components.** The hepatic (bilirubin), coagulation (platelets), and renal (creatinine) subscores retain the original Sepsis-3/SOFA cut-points without modification. Two exceptions apply to the renal subscore: the urine-output criterion is omitted because it is not routinely recorded on general wards, and patients with end-stage renal disease (ESRD) are assigned the maximum renal subscore of 4 points.

**Score computation.** The admission SOFA score will be obtained as the sum of the six organ-system subscores. In keeping with the original SOFA concept, points are attributed only to new or worsening abnormalities relative to the patient's pre-admission baseline.

**Reference and study-specific scoring tables.** Box 1 reproduces the original SOFA scoring system; Box 2 summarizes the modified SOFA (mSOFA) framework of Raymond et al.; and Box 3 presents the final study-specific operationalization as it will be applied in this trial.

**Handling of missing data.** For laboratory variables contributing to the SOFA calculation, when no value is recorded on the day of admission, the most recent measurement within the preceding 30-day window will be carried forward using a last-observation-carried-forward (LOCF) approach. The choice of a 30-day window is consistent with the timeframe endorsed by the European Society of Anaesthesiology and Intensive Care (ESAIC) for the validity of preoperative assessment in non-ICU adult patients [Lamperti et al., 2025]. Any components that remain missing after this step will be assigned the normal-range (0-point) value, in keeping with the convention adopted in the Sepsis-3 consensus framework [Singer et al., 2016].

Box 1. Original SOFA scoring system

|  | **Cardiovascular** | **Respiratory** | **CNS** | **Creatinine** | **Platelets** | **Bilirubin** |
| --- | --- | --- | --- | --- | --- | --- |
| **Score** | **Mean arterial pressure (mmHg)** | **PaO₂/FiO₂** | **GCS** | **mg/dL** | **× 10^9^/L** | **mg/dL** |
| 0 | MAP ≥70 | ≥400 | 15 | <1.2 | ≥150 | <1.2 |
| 1 | MAP <70 | <400 | 13-14 | 1.2-1.9 | <150 | 1.2-1.9 |
| 2 | Dopamine <5 or dobutamine (any dose) | <300 | 10-12 | 2.0-3.4 | <100 | 2.0-5.9 |
| 3 | Dopamine 5.1-15 or epinephrine ≤0.1 or norepinephrine ≤0.1 | <200 with respiratory support | 6-9 | 3.5-4.9 | <50 | 6.0-11.9 |
| 4 | Dopamine >15 or epinephrine >0.1 or norepinephrine >0.1 | <100 with respiratory support | <6 | ≥5.0 | <20 | >12.0 |

PaO2, Partial pressure of oxygen; FiO2, Fraction of inspired oxygen; CNS, Central Nervous System; GCS, Glasgow Coma Score

Box 2. Modified SOFA (mSOFA) scoring system proposed by Raymond et al.

|  | Cardiovascular | Respiratory | CNS | Creatinine | Platelets | Bilirubin |
| --- | --- | --- | --- | --- | --- | --- |
| Score | Systolic BP (mmHg) | Oxygen Saturation (%) | GCS | μmol/L | × 10^9^/L | μmol/L |
| 0 | BP ≥100 | ≥90% pre-O_2_ | 15 | <110 | ≥150 | <20 |
| 1 | BP <100,  pre-IV fluids | <90% pre- O_2_ therapies | 13-14 | 110-170 | <150 | 20-32 |
| 2 | BP <100,  post-IV fluids | <90% despite O_2_ therapies | 10-12 | 171-299 | <100 | 33-101 |
| 3 | Inotropes | Ventilated | 6-9 | 300-400 | <50 | 102-204 |
| 4 |  |  | <6 | >400 | <20 | >204 |

CNS, Central Nervous System; GCS, Glasgow Coma Score; BP, Blood Pressure

Box 3. Study-specific operationalization of the modified SOFA (mSOFA) used in this trial

|  | Cardiovascular | Respiratory | CNS | Creatinine | Platelets | Bilirubin |
| --- | --- | --- | --- | --- | --- | --- |
| Score | Systolic BP (mmHg) | Oxygen Saturation (%) | AVPU | mg/dL | × 10^9^/L | mg/dL |
| 0 | BP ≥100 | ≥90% pre-O_2_ | A | <1.2 | ≥150 | <1.2 |
| 1 | BP <100,  pre-IV fluids | <90% pre- O_2_ therapies | V/P/U | 1.2-1.9 | <150 | 1.2-1.9 |
| 2 | BP <100,  post-IV fluids | <90% despite O_2_ therapies |  | 2.0-3.4 | <100 | 2.0-5.9 |
| 3 | Inotropes | Ventilated |  | 3.5-4.9 | <50 | 6.0-11.9 |
| 4 |  |  |  | ≥5.0 | <20 | >12.0 |

AVPU, Alert / Verbal / Pain / Unresponsive; ESRD, end-stage renal disease (assigned the maximum renal subscore of 4 points); BP, blood pressure; CNS, central nervous system

**Supplementary Methods 2. Operational definition of sepsis based on ASE criteria**

The Centers for Disease Control and Prevention (CDC) developed the Adult Sepsis Event (ASE) definition as an EHR-extractable surveillance construct that operationalizes the Sepsis-3 conceptual framework into a reproducible case-finding algorithm [Rhee et al., 2017]. By anchoring sepsis ascertainment to two routinely captured EHR signals—blood-culture sampling and antimicrobial administration—coupled with objectively measurable organ-dysfunction events, the ASE definition enables automated, standardized identification of sepsis events at scale. In this trial, the prespecified sepsis subgroup will be identified using this ASE framework, applied to the EHR data captured during each patient's index hospitalization.

A direct application of the Sepsis-3 definition is not adopted for this subgroup analysis. Whereas the admission mSOFA score (Methods 1) is required only at a single time point and can therefore be reconstructed from ward-level data, a SOFA-based Sepsis-3 operationalization requires longitudinally measured SOFA components to detect an acute ≥2-point rise—a requirement that ward EMR data cannot reliably support owing to non-daily laboratory sampling. Although Sepsis-3 is authoritative as a clinical construct, its operational use requires a serially measured SOFA score; on general wards, the laboratory components of SOFA are not obtained on a daily basis, and the resulting missingness precludes reliable, automated case-finding. The ASE framework was specifically designed to address this limitation by substituting the SOFA-based organ-dysfunction signal with surrogates that are reliably captured during routine ward care. Accordingly, the ASE definition will serve as the operational standard for this trial, with study-specific implementation details described below.

**Operationalization and Scoring**

**Presumed infection.** Presumed infection will be ascertained when systemic antimicrobial therapy is initiated for ≥4 qualifying antimicrobial days (QADs) within a window spanning the 2 calendar days before to the 2 calendar days after a blood-culture collection. A QAD is defined as any calendar day on which a systemic antimicrobial is administered, and an antimicrobial is considered new if it has not been administered on either of the two preceding calendar days.

**Concurrent organ dysfunction.** Within the same ±2-day blood-culture window, organ dysfunction will be ascertained when at least one of the following events occurs: (i) initiation of a new vasopressor infusion (norepinephrine, dopamine, epinephrine, phenylephrine, or vasopressin); (ii) initiation of invasive mechanical ventilation; (iii) doubling of serum creatinine, or a ≥50% reduction in estimated glomerular filtration rate (eGFR) relative to baseline, in patients without end-stage renal disease; (iv) total bilirubin ≥2.0 mg/dL with a ≥100% rise from baseline; (v) platelet count <100 × 10⁹/L with a ≥50% drop from baseline; or (vi) serum lactate ≥2.0 mmol/L.

**Baseline organ function.** Baseline organ dysfunction will be operationalized as the most favorable value of each variable observed throughout the index hospitalization.

**Sepsis onset date.** The sepsis onset date will be assigned to the earliest calendar day, within the ±2-day blood-culture window, on which any of the three qualifying events—blood-culture collection, the first QAD, or fulfillment of any organ-dysfunction criterion—is met. As an illustration, in a patient whose first QAD is recorded on hospital day 1, whose blood culture is drawn on hospital day 2, and who is intubated on hospital day 4, hospital day 1 is recorded as the sepsis onset date.

**Handling of missing data.** For ASE ascertainment, missing values for any criterion will be treated as absence of that criterion (scored as 0). Forward-filling of laboratory or physiologic measurements will not be applied, consistent with the original ASE specification [Rhee et al., 2017].

**Supplementary References**

Singer M, Deutschman CS, Seymour CW, Shankar-Hari M, Annane D, Bauer M, Bernard GR, Chiche J-D, Coopersmith CM, Hotchkiss RS, Levy MM, Marshall JC, Martin GS, Opal SM, Rubenfeld GD, van der Poll T, Vincent J-L, Angus DC. The Third International Consensus Definitions for Sepsis and Septic Shock (Sepsis-3). JAMA 2016 Feb 23;315(8):801–810. PMID:26903338

Raymond NJ, Nguyen M, Allmark S, Woods L, Peckler B. Modified Sequential Organ Failure Assessment sepsis score in an emergency department setting: retrospective assessment of prognostic value. Emerg Med Australas 2019 Jun;31(3):339–346. PMID:30109767

Lamperti M, Romero CS, Guarracino F, Cammarota G, Vetrugno L, Tufegdzic B, Lozsan F, Macias Frias JJ, Duma A, Bock M, Ruetzler K, Mulero S, Reuter DA, La Via L, Rauch S, Sorbello M, Afshari A. Preoperative assessment of adults undergoing elective noncardiac surgery: Updated guidelines from the European Society of Anaesthesiology and Intensive Care. Eur J Anaesthesiol 2025 Jan 1;42(1):1–35. PMID:39492705

Rhee C, Dantes R, Epstein L, Murphy DJ, Seymour CW, Iwashyna TJ, Kadri SS, Angus DC, Danner RM, Fiore AE, Jernigan JA, Martin GS, Septimus E, Warren DK, Karcz A, Chan C, Menchaca JT, Wang R, Gruber S, Klompas M; CDC Prevention Epicenter Program. Incidence and Trends of Sepsis in US Hospitals Using Clinical vs Claims Data, 2009–2014. JAMA 2017 Oct 3;318(13):1241–1249. PMID:28903154
