# Supplementary material for: Clinical Effectiveness of AI-Based Cardiac Arrest Prediction System for General Ward Patients (VITAL-MD): Protocol for a Multicenter Pragmatic Stepped Wedge Cluster Randomized Controlled Trial
Source: JMIR Res Protoc. 2026 Oct 19;15:e104230. doi: 10.2196/104230 (PMC13679675; doi:10.2196/104230)
Supplement: Supplementary file 4 [file resprot-v15-e104230-s004.docx]

Statistical Analysis Plan (SAP)

**Protocol Title:**

Clinical effectiveness of artificial intelligence-based prediction model for cardiac arrest in general ward-admitted patients (VITAL-MD): a structured study protocol for multicenter pragmatic cluster randomized trial

**Sponsor Name:**

Inha University Hospital

This is the Statistical Analysis Plan (SAP) for the VITAL-MD trial. This document may be updated as necessary throughout the course of the study.

| Version No. | Valid from (date of implementation) | Replacement of | Number of pages |
| --- | --- | --- | --- |
| 1.0 | Apr 1, 2025 | N/A | 20 |

I confirm that I have read and approved the above-referenced document for use in the VITAL-MD trial.

Written by:

| Organization | Name | Date |
| --- | --- | --- |
| [CK Insights] | [Kang Woo Suk] | 2025-2-21 |

Signature: _______________________

Reviewed by:

| Organization | Name | Date |
| --- | --- | --- |
| [Inha University] | [Jung Soo Kim] | 2025-3-1 |

Signature: _______________________

**TABLE OF CONTENTS**

[**Glossary of abbreviations**	4](#_Toc199406257)

[**1. STUDY OVERVIEW**	5](#_Toc199406258)

[**1.1 Study Design and Randomization**	6](#_Toc199406259)

[**1.2 Study Objectives**	8](#_Toc199406260)

[**1.2.1 Primary Objective**	8](#_Toc199406261)

[**1.2.2 Secondary Objectives**	8](#_Toc199406262)

[**1.3 Study Population**	8](#_Toc199406263)

[**1.3.1 Inclusion Criteria**	8](#_Toc199406264)

[**1.3.2 Exclusion Criteria**	9](#_Toc199406265)

[**1.4 Study Outcomes**	9](#_Toc199406266)

[**1.4.1 Primary Outcome**	9](#_Toc199406267)

[**1.4.2 Secondary Outcomes**	9](#_Toc199406268)

[**1.4.3 Overview of Outcomes**	9](#_Toc199406269)

[**1.5 Sample Size Calculation**	9](#_Toc199406270)

[**1.6 Study Procedures**	10](#_Toc199406271)

[**1.6.1 Procedures**	10](#_Toc199406272)

[**2. STATISTICAL METHODOLOGY**	11](#_Toc199406273)

[**2.1 Statistical Variables**	11](#_Toc199406274)

[**2.1.1 Background and Demographic Characteristics**	11](#_Toc199406275)

[**2.1.2 Confounding Factors**	11](#_Toc199406276)

[**2.1.3 Effectivenss**	11](#_Toc199406277)

[**2.1.4 Safety**	11](#_Toc199406278)

[**2.2 Statistical Analysis Populations**	12](#_Toc199406279)

[**2.2.1 Analysis of Crossover Patients**	13](#_Toc199406280)

[**2.2.2 Handling of AI-SaMD Withdrawal or Deactivation**	13](#_Toc199406281)

[**2.2.3 Compliance Analysis**	13](#_Toc199406282)

[**2.3 Statistical Methods**	13](#_Toc199406283)

[**2.3.1 Demography and Baseline Patient Characteristics**	13](#_Toc199406284)

[**2.3.2 Analysis of Effectiveness Outcomes**	14](#_Toc199406285)

[**2.3.2.1 Primary analysis**	14](#_Toc199406286)

[**2.3.2.2 Secondary analysis**	14](#_Toc199406287)

[**2.3.2.3 Subgroup analysis**	14](#_Toc199406288)

[**2.3.3 Sensitivity analysis**	15](#_Toc199406289)

[**2.3.4 Statistical Interim Analyses**	15](#_Toc199406290)

[**2.4 Data Processing Conventions**	16](#_Toc199406291)

[**2.4.1 Definition of Baseline**	16](#_Toc199406292)

[**2.4.2 Missing Data**	16](#_Toc199406293)

[**2.4.3 Time Window**	17](#_Toc199406294)

[**2.4.4 Unscheduled Visits**	17](#_Toc199406295)

[**2.4.5 Centers Pooling**	17](#_Toc199406296)

[**3. STATISTICAL ANALYSIS SOFTWARE**	17](#_Toc199406297)

[**4. References**	18](#_Toc199406298)

# **Glossary of abbreviations**

| **Abbreviation** | **Full Term** |
| --- | --- |
| AI | artificial intelligence |
| AI-SaMD | artificial intelligence-based software as a medical device |
| ARD | adjusted risk difference |
| ARR | adjusted relative risk |
| BT | body temperature |
| CCI | charlson comorbidity index |
| CI | confidence interval |
| CPC | cerebral performance category |
| DBP | diastolic blood pressure |
| DE | design effect |
| DeepCARS^®^ | VUNO Med^®^-DeepCARS^®^ |
| DNR | do-not-resuscitate |
| DSMB | data and safety monitoring board |
| EMR | electronic medical record |
| EWS | early warning system |
| FAS | full analysis set |
| GLMM | generalized linear mixed model |
| HR | heart rate |
| ICC | intra-cluster correlation coefficient |
| ICU | intensive care unit |
| IHCA | in-hospital cardiac arrest |
| IQR | interquartile range |
| IRB | institutional review board |
| ITT | intention-to-treat |
| LMM | linear mixed model |
| LOCF | last observation carried forward |
| MEWS | modified early warning score |
| MFDS | ministry of food and drug safety |
| NEWS | national early warning score |
| PI | principal investigator |
| PPS | per protocol set |
| PSM | propensity score matching |
| RCT | randomized controlled trial |
| RRS | rapid response system |
| SAP | statistical analysis plan |
| SBP | systolic blood pressure |
| SD | standard deviation |
| SOFA | sequential organ failure assessment |
| SS | safety set |
| SW-CRT | stepped-wedge cluster randomized trial |
| UIT | unplanned intensive care unit transfer |

# **1. STUDY OVERVIEW**

This Statistical Analysis Plan (SAP) is based on a multicenter Stepped-Wedge Cluster Randomized Trial (SW-CRT) designed to evaluate the clinical effectiveness of an artificial intelligence (AI)-based predictive model, VUNO Med^®^-DeepCARS^®^ (DeepCARS^®^) (version 1.3.2), for detecting in-hospital cardiac arrest (IHCA) in general ward patients.

The objective of this study is to objectively assess the impact of sequentially implementing the DeepCARS^®^ system in real-world clinical settings, compared to conventional Early Warning System (EWS)-based care, in preventing general ward cardiac arrest. DeepCARS^®^ is an artificial intelligence-based Software as a Medical Device (AI-SaMD) that utilizes patients’ vital sign data to provide real-time predictions of cardiac arrest risk in general wards.

This trial adopts a randomized stepped-wedge structure (Figure 1). Four tertiary hospitals are designated as clusters, each of which transitions to the intervention phase at a predefined randomized time point. Initially, all hospitals begin in the control phase (without the use of AI-SaMD), and transition sequentially to the intervention phase according to a randomized schedule. A 7-day washout period is applied at each transition to allow for stabilization of the intervention effect.

With this SW-CRT design, each hospital functions as both control and intervention over time, enabling control of inter-hospital heterogeneity and adjustment for temporal trends. Thus, the study is conducted based on a pragmatic and ethically sound design to evaluate the real-world clinical effectiveness of the AI-SaMD (Figure 2).

Figure 1. Schematic diagram of the stepped-wedge randomized design.

The diagram illustrates the sequence of pre-implementation, transitional (washout), and post-implementation periods. Each wedge spans 2.4 months and includes a 7-day transitional period.

Figure 2. Flowchart of the study design.

The flowchart illustrates the study design, including screening, enrollment, allocation, intervention, follow-up, and analysis for the AI-SaMD-guided and usual care cohorts, as well as the target and non-target cohorts.

## **1.1 Study Design and Randomization**

This study is designed as a SW-CRT, in which four participating hospitals are assigned as individual clusters and randomly allocated to different intervention initiation time points (Figure 1) (1). The entire study duration spans 12 months, during which each hospital sequentially transitions to the intervention phase at intervals of approximately 2.4 months. Initially, all hospitals begin in the control phase (usual care), and then transition to the intervention phase at pre-randomized time points, during which the AI-SaMD is implemented. The transition order of each cluster (hospital) is determined in advance via a centralized randomization procedure prior to study initiation. Each hospital is notified of its scheduled transition point one month prior to the actual transition. A 7-day washout period is applied immediately after each transition, and patients enrolled during this period are analyzed separately. This design allows for the control of structural differences between hospitals and adjustment for temporal confounding, thereby enabling a more accurate assessment of the intervention effect of the system.

The intervention phase is defined as the period during which the AI-SaMD is fully integrated into the hospital’s Electronic Medical Record (EMR) system, and real-time risk alerts are actively delivered to clinical staff. The control phase refers to the period during which the system is not operational, and care is guided solely by conventional EWSs such as the National Early Warning Score (NEWS), Modified Early Warning Score (MEWS), or single-parameter track and trigger system (SPTTS) (2).

The detailed study design is presented in Figure 2. The effectiveness of the intervention will be evaluated by comparing patient outcomes between the intervention (post-implementation) and the control groups (pre-implementation periods). The primary analysis will focus on patients at a high risk of general ward cardiac arrest or acute deterioration, defined as those who exceed the AI-SaMD alert threshold at least once during their general ward hospitalization (target cohort). Because the target cohort corresponds to the intended use of the AI-SaMD, it is prespecified as the primary analysis population (3-7). Given the very low incidence of the primary outcome among non-high-risk patients, designating the target cohort as the primary analysis population is justified and consistent with prior studies (1, 8-12). Patients who do not exceed the alert threshold will be classified into non-target cohort. Patients who do not exceed the alert threshold will be classified into non-target cohort. To validate the restriction of study groups to the target cohort, patient outcomes will also be compared between the target and non-target cohorts in a secondary analysis.

The AI-SaMD score is computed using four vital sign parameters: blood pressure, heart rate, respiratory rate, and body temperature (3-7). The alert thresholds and notification rules may differ by hospital depending on site-specific operational settings.

## **1.2 Study Objectives**

### **1.2.1 Primary Objective**

To evaluate the impact of implementing the AI-SaMD on the incidence of cardiac arrest among general ward patients.

### **1.2.2 Secondary Objectives**

To evaluate the impact of implementing the AI-SaMD on the following outcomes among general ward patients:

- All-cause in-hospital mortality
- Length of hospital stay
- Length of intensive care unit (ICU) stay
- Time to unplanned intensive care unit transfer (UIT) after the first alert
- Cerebral Performance Category (CPC) score at discharge among patients who experienced general ward cardiac arrest

## **1.3 Study Population**

### **1.3.1 Inclusion Criteria**

The inclusion criteria for participating hospitals and eligible patients are as follows:

**A. Hospital Selection Criteria**

1. Hospitals with a similar inpatient capacity (approximately 850 inpatient beds).
2. Hospitals with comparable Rapid Response System (RRS) operating hours.
3. Hospitals capable of operating the AI-SaMD through integration with their EMR systems. Hospitals that have already implemented the AI-SaMD are excluded.

**B. Patient Inclusion Criteria**

1. Adult patients aged 19 years or older (≥19 years) admitted to general wards.

### **1.3.2 Exclusion Criteria**

Patients meeting any of the following criteria will be excluded from the study:

1. Pediatric patients under the age of 19 (<19 years).
2. Cases in which all predictor variables required to compute the AI-SaMD score—systolic/diastolic blood pressure (SBP/DBP), heart rate (HR), body temperature (BT), and respiratory rate (RR)—are missing, making score calculation impossible.

※ Note: The AI-SaMD (DeepCARS^®^) is approved by the Ministry of Food and Drug Safety (MFDS) in the Republic of Korea for score calculation even when only a single vital sign is available. Therefore, partial missing data do not constitute exclusion criteria.

## **1.4 Study Outcomes**

### **1.4.1 Primary Outcome**

Incidence of cardiac arrest among general ward patients

### **1.4.2 Secondary Outcomes**

- All-cause in-hospital mortality
- Length of hospital stay
- Length of ICU stay
- Time to UIT after the first alert
- CPC score at discharge among patients who experienced general ward cardiac arrest

### **1.4.3 Overview of Outcomes**

The outcome variables are classified as binary, continuous, or time-to-event variables, and appropriate statistical models will be applied accordingly.

## **1.5 Sample Size Calculation**

The primary hypothesis is that implementing the AI-SaMD will significantly reduce the incidence of cardiac arrest in patients admitted to the general ward, compared with usual care.

- Null hypothesis ($H_{0}$): There is no difference in the incidence of general ward cardiac arrest between the intervention and control groups in the target cohort.
- Alternative hypothesis ($H_{1}$): There is a difference in the incidence of general ward cardiac arrest between the intervention and control groups in the target cohort.

The general ward cardiac arrest incidence among high-risk patients by the AI-SaMD (target cohort) in the control group is assumed to be 6.70%, based on our prior studies (3). Given prior trials reporting an average 40% reduction in clinical deterioration within the target cohort, the expected general ward cardiac arrest incidence in the intervention group is 4.00% (1, 8, 13). The significance level ($\alpha$) is set at 0.05 (two-sided), with a statistical power ($1-\beta$) of 0.80. The intra-cluster correlation coefficient (ICC) is set at 0.01, based on an RRS meta-analysis (14). Each cluster-period is expected to enroll approximately 350 patients, based on our prior study (3, 4, 6).

For the SW-CRT, the required sample size is calculated as (15):

$$n=n_{RCT}\times(B+JT)\times CF$$

$$n_{RCT}=2\times\frac{\left( z_{1-\alpha/2}\cdot\sqrt{2\bar{p}\left( 1-\bar{p} \right)}+z_{1-\beta}\cdot\sqrt{p_{1}\left( 1-p_{1} \right)+p_{0}\left( 1-p_{0} \right)} \right)^{2}}{\left( p_{0}-p_{1} \right)^{2}}$$

$$CF=\frac{1+\rho(JTK+BK-1)}{1+\rho(\frac{1}{2}JTK+BK-1)}\frac{3(1-\rho)}{2T(J-\frac{1}{J})}$$

where,

- $n_{RCT}$: the total required sample size for an individually randomized controlled trial (RCT)
- $CF$: the correction factor for the stepped-wedge design
- $p_{0}=0.067$ (the expected general ward cardiac arrest incidence in the control group)
- $p_{1}=0.040$ (assumed general ward cardiac arrest incidence in the intervention group)
- $\bar{p}=(p_{0}+p_{1})/2$
- $z_{1-\alpha/2}=1.96$ ($\alpha=0.05$, two-sided)
- $z_{1-\beta}=0.84$ (power ($1-\beta)=0.80$)
- $B=1$ (the number of baseline measurement periods)
- $J=4$ (the number of crossover points at which clusters switch to the intervention)
- $T=1$ (the number of measurement periods within each crossover step)
- $K=350$ (the number of patients per cluster-period)
- $\rho=0.01$ (ICC)

The required sample size is 7,002 patients in the target cohort. Assuming the AI-SaMD alerts 10% of patients as high risk and allowing for a 5% dropout, the total enrollment needed is estimated at 72,695 (3, 4, 6). The non-target cohort is estimated at 65,693.

## **1.6 Study Procedures**

Each hospital will transition to the intervention phase based on a pre-randomized time point. All patient data collected after the hospital's transition point will be considered part of the intervention group.

Each patient will be assigned to either the intervention (post-implementation) or control (pre-implementation) group based on their admission date relative to the hospital’s intervention implementation date.

Patient-level data, including baseline characteristics, daily vital signs, AI-SaMD scores, activated intervention, cardiac arrest, mortality, and ICU records, will be collected using each hospital’s EMR system.

### **1.6.1 Procedures**

All necessary procedures documented in this study are as follows (Table 1).

Table 1. Schedule of procedures during study period

| Procedure | Study period | | |
| --- | --- | --- | --- |
|  | Admission | General ward stay | Discharge |
| Screening/Enrollment/Allocation |  |  |  |
| Eligibility screening | O | - | - |
| Exclusion criteria | O | O | O |
| Randomization | O | - | - |
| Intervention |  |  |  |
| Intervention (AI-SaMD) | - | O | - |
| Comparator (Usual care) | - | O | - |
| Follow-up (Data collection) |  |  |  |
| Baseline characteristics | O | - | - |
| Daily vital signs | O | O | O |
| Intervention records | - | O | - |
| General ward cardiac arrest | - | O | - |
| All-cause in-hospital mortality | - | - | O |
| Hospital length of stay | - | - | O |
| Total ICU length of stay | - | O | - |
| Time to UIT after the first alert | - | O | - |
| Cerebral Performance Category | - | - | O |

AI-SaMD, artificial intelligence-software as a medical device; ICU, intensive care unit; UIT, unplanned intensive care unit transfer.

# **2. STATISTICAL METHODOLOGY**

## **2.1 Statistical Variables**

In this study, outcome variables are categorized as binary, continuous, or time-to-event variables, and appropriate statistical methods will be applied according to the nature of each variable. The primary outcome is the incidence of cardiac arrest in general ward patients. Secondary outcomes include all-cause in-hospital mortality, hospital length of stay, ICU length of stay, time to UIT, and CPC score.

### **2.1.1 Background and Demographic Characteristics**

Basic demographic and background characteristics such as age, sex, and type of hospital admission will be summarized using descriptive statistics to assess the baseline balance between the intervention and control groups. This is intended to identify any potential differences in baseline characteristics that may influence the interpretation of the primary analysis results.

- **Continuous variables:** It will be presented as mean ± standard deviation (SD) or median with interquartile range (IQR).
- **Categorical variables:** It will be presented as frequency (n) and percentage (%).

### **2.1.2 Confounding Factors**

In the primary analysis, key confounders will include demographics such as age, sex, department (internal medicine or surgical), day of admission (weekday or weekend), and seasonality (spring, summer, fall, or winter) and clinically relevant variables such as the NEWS, sequential organ failure assessment (SOFA), Charlson comorbidity index (CCI), number of staff, bed occupancy rate, and secular time trends. These variables will be included as covariates in the main statistical model to minimize potential confounding and enhance the accuracy of the effect estimates.

### **2.1.3 Effectiveness**

The effectiveness of the AI-SaMD system will be evaluated by assessing its impact on the reduction of cardiac arrest incidence in general ward patients.

Differences between the intervention and control groups will be analyzed using generalized linear mixed models (GLMM), incorporating both time-period effects and hospital-level cluster effects (16). This approach is intended to ensure statistical validity by reflecting the characteristics of the stepped-wedge cluster randomized trial design.

### **2.1.4 Safety**

AI-SaMD is a predictive system designed to prompt early clinical responses by medical staff and does not involve any direct physiological interventions. However, the potential for indirect harms—such as unnecessary clinical interventions or UIT triggered by alerts—should be carefully considered.

Accordingly, UIT, prolonged hospital stays, and other indicators of possible over-intervention occurring during the study period will be monitored as secondary safety outcomes and assessed using descriptive statistical analysis.

## **2.2 Statistical Analysis Populations**

The analysis population in this study is defined based on the intended clinical use of the AI-SaMD and the characteristics of the SW-CRT design.

The primary analysis population will consist of patients admitted to ge who, at any point during their hospitalization, record an AI-SaMD score that meets or exceeds the predefined alert threshold (Target cohort). These patients represent the subset for whom the structural conditions for system-triggered alerts are satisfied and are considered the most appropriate group for evaluating the direct clinical impact of the intervention.

Patients whose AI-SaMD scores never reach the alert threshold will be classified as the Non-target cohort. They will be excluded from the primary analysis but may be included in sensitivity and secondary analyses to assess the robustness and generalizability of the intervention effect.

In addition, crossover patients—those admitted during the washout period and who may have been exposed to both control and intervention phases—will be excluded from the primary analysis or included only in sensitivity analyses to evaluate their potential impact.

This population definition is designed to minimize bias in estimating the intervention effect and to reflect the actual clinical utility of the AI-SaMD.

- **Summary of Analysis Populations**
  - Target cohort: Patients who record at least one AI-SaMD score meeting or exceeding the alert threshold during hospitalization.
  - Non-target cohort: Patients who do not trigger any AI-SaMD alerts during hospitalization.
  - Crossover patients: Patients admitted during the washout period who are exposed to both control and intervention phases. These patients are excluded from the primary analysis, but included in sensitivity analyses, as appropriate.
- **Full Analysis Set (FAS)**: Includes all eligible patients admitted during the pre- or post-implementation periods as determined by the predefined randomized transition schedule at each hospital. Since randomization occurs at the cluster (hospital) level, patients are automatically assigned to the control or intervention group based on their admission date relative to the site-specific implementation date. The primary analysis will follow the intention-to-treat (ITT) principle.
- **Per Protocol Set (PPS)**: Includes patients who are admitted within the defined pre- and post-implementation periods without major protocol violations. This set reflects patients for whom the intervention is appropriately applied, and will be used for per-protocol analysis.

### **2.2.1 Analysis of Crossover Patients**

Patients admitted during the washout period who are exposed to both the control and intervention phases (crossover patients) will be excluded from the primary analysis. However, they will be included in sensitivity analyses to assess the robustness of the results and examine the impact of their inclusion.

This approach is intended to ensure clarity in group assignment and to minimize potential confounding of the intervention effect.

### **2.2.2 Handling of AI-SaMD Withdrawal or Deactivation**

Temporary discontinuation of the AI-SaMD at the hospital level or failure to apply the system to individual patients (e.g., due to technical errors or clinical decisions) will be considered as non-application cases. These cases will be clearly flagged in the dataset.

Such cases may be excluded from the primary analysis. However, they will be included in supplementary analyses to evaluate the sensitivity of the results to system non-application and to assess the consistency and effectiveness of the intervention.

### **2.2.3 Compliance Analysis**

Whether clinical staff responded to the AI-SaMD alerts with actual clinical actions will be evaluated as a supplementary analysis of compliance. This assessment aims to indirectly determine whether the alerts led to changes in patient management beyond mere system activation.

The analysis will summarize the association between outcomes and alert compliance rate or alert response time. It will not be included in the primary effectiveness analysis.

## **2.3 Statistical Methods**

This section outlines the statistical analysis methods and their applications for this study. This SAP serves as a practical guide for performing the statistical analyses and all analyses shall be conducted in accordance with the methods described herein.

### **2.3.1 Demography and Baseline Patient Characteristics**

Demographic and clinical characteristics at the time of admission will be summarized using descriptive statistics to assess the balance between the intervention and control groups.

- **Continuous variables:** It will be presented as mean ± SD or as median with IQR, depending on the distribution.
- **Categorical variables:** It will be summarized using frequencies (n) and percentages (%).

### **2.3.2 Analysis of Effectiveness Outcomes**

The primary analysis will evaluate the incidence of cardiac arrest among general ward patients as the primary outcome. Secondary outcomes will include all-cause in-hospital mortality, length of hospital stay, length of ICU stay, time to UIT after the first alert, and CPC score at discharge among patients who experienced general ward cardiac arrest.

All outcomes will be analyzed using GLMM appropriate for each outcome type: binary outcomes will be reported as adjusted relative risks (ARR) with 95% confidence intervals (CI), and continuous outcomes as adjusted risk difference (ARD) with 95% CIs (16). The model will include intervention status (intervention vs. control), defined by the hospital’s transition schedule, as the main predictor, and will adjust for time period and cluster (hospital) effects, as well as prespecified patient-level covariates: age, sex, department (internal medicine or surgical), season (spring, summer, fall, winter), day of admission (weekday or weekend), NEWS, SOFA, CCI, number of staff, bed occupancy rate, and secular time trends. To ensure valid inference given the small number of clusters, small-sample corrections (between-within degrees-of-freedom) will be applied (17). Because only four hospitals were randomized, the intervention effect will be evaluated using a 1-degree-of-freedom Wald F test with the Between-Within degrees-of-freedom approximation reflecting the multiple-period stepped-wedge design. The denominator degrees of freedom will be specified as $(I-1)J$, where $I$ is the number of randomized hospitals and $J$ is the number of study periods; corresponding 95% confidence intervals will use the t distribution with the same degrees of freedom.

### **2.3.3 Secondary analysis**

### ***2.3.3.1 Non-target cohort analysis***

Patients who do not exceed the predefined alert threshold during their general ward admission will be classified into the non-target cohort. To validate the restriction of the primary analysis to the target cohort, a secondary analysis will compare outcomes between the target and non-target cohorts. In addition, a full-population analysis will be conducted using the same modeling framework as the primary analysis to estimate the hospital-wide intervention effect.

Baseline characteristics, including the alert distribution, will be summarized descriptively and compared. The outcome comparison will include the primary and secondary outcomes, analyzed using the same outcome-specific models as in the primary analysis. The model will include alert status (target vs. non-target), defined by the AI-SaMD alert threshold, as the main predictor, and will adjust for time period and cluster (hospital) effects, as well as prespecified patient-level covariates consistent with the primary analysis.

The results will provide complementary evidence regarding the validity of focusing the primary analysis on the target cohort.

### ***2.3.3.2 Survival analysis***

Time-to-event outcomes will be descriptively summarized using Kaplan–Meier estimates to visualize event time to cardiac arrest and all-cause in-hospital mortality. Kaplan-Meier methods will be used to estimate survival functions, and differences between groups will be assessed with the log-rank test (17, 18).

### ***2.3.3.3 Subgroup analysis***

Subgroup analysis will be performed to evaluate whether the intervention effect of the AI-SaMD varies across clinically relevant patient and contextual factors. Predefined subgroups will include patient characteristics, clinical contexts, or reasons and types of interventions in response to the AI-SaMD alert. The effectiveness of the AI-SaMD will be assessed within each subgroup to explore its consistency and potential clinical utility across different settings.

### ***2.3.3.4 Lead-time analysis***

Lead-time analyses will evaluate the interval from AI-SaMD alert to RRS response or clinical intervention and its association with patient outcomes. Temporal changes in lead time after implementation will also be assessed to examine staff response patterns and adaptation to the AI-SaMD–guided workflow.

### **2.3.4 Sensitivity Analysis**

Sensitivity analyses will be conducted as supplementary evaluations to assess the statistical robustness of the primary and secondary outcomes and to examine the consistency of the intervention effect.

The following sensitivity analyses are planned:

- Unadjusted analysis
  - To evaluate the intervention effect without adjusting for confounding variables.
  - Primary and secondary outcomes will be estimated using an unadjusted regression model.
- Propensity score matching (PSM) (20)
  - To evaluate the intervention effect after balancing covariates between the intervention and control groups.
  - A 1:1 matching based on propensity scores will be performed, and the primary outcomes will be estimated accordingly.
- Crossover patients
  - To evaluate the impact of including or excluding crossover patients (i.e., patients admitted during the washout period who are exposed to both control and intervention phases).

### **2.3.5 Statistical Interim Analyses**

In alignment with the study protocol and reflecting the characteristics of the SW-CRT design, an interim analysis is planned. The primary purpose of this analysis is to monitor for any unexpected and substantial increase in the incidence of general ward cardiac arrest following the implementation of the AI-SaMD, thereby ensuring the ethical and clinical safety of the trial.

Interim analyses will be conducted at three time points, excluding the first cluster transition. The analysis will focus on the general ward cardiac arrest incidence rate, comparing the pre-implementation period at each hospital with the same calendar month of the previous year. The primary metric will be the number of general ward cardiac arrest events per 1,000 admissions.

Basic adjustments for potential confounders will be made, and emphasis will be placed on identifying clinically significant trends rather than formal hypothesis testing. If the general ward cardiac arrest incidence rate increases by more than 50% compared to the same period in the previous year, an independent statistician will report the findings to the Data and Safety Monitoring Board (DSMB). The DSMB will review and interpret the data and provide recommendations to the Institutional Review Board (IRB) and the Principal Investigator (PI) regarding the continuation of the study.

This SAP does not include any formal statistical hypothesis testing for interim analyses. Instead, results will be presented descriptively and used for informal trend monitoring. All analyses will be performed by the designated statistician or data manager according to predefined analytic code. Reports provided to the DSMB will consist of numerical summaries and graphical evaluations of temporal trends.

- Summary of Interim Analysis Plan:
  - Timing: At three transition points (excluding the first cluster transition)
  - Outcome: General ward cardiac arrest incidence (per 1,000 admissions)
  - Comparison: Pre-implementation period vs. same month in the previous year
  - Adjustment: Basic confounding factor adjustment
  - Analysis Guideline: Analyses are solely for DSMB review; no formal hypothesis testing
  - Follow-up Action: If a ≥50% increase in general ward cardiac arrest is observed, DSMB discussion → Recommendation to IRB and PI

## **2.4 Data Processing Conventions**

This section defines key data handling rules including the timing of baseline assessment, handling of missing data, and determination of observation windows. These principles are established to ensure consistency and transparency in the conduct of statistical analyses.

### **2.4.1 Definition of Baseline**

The baseline is defined as the date of admission on which the patient first recorded an AI-SaMD score. Baseline information will be based on values available as of this admission date.

### **2.4.2 Missing Data**

For missing data related to the calculation of the AI-SaMD score, the system applies an internal Last Observation Carried Forward (LOCF) mechanism. In cases where a patient’s vital sign is partially missing, the system automatically uses the most recently recorded value to compute the score. This imputation is handled within the device software and does not require additional statistical adjustment during the analysis.

For key outcome variables (e.g., general ward cardiac arrest, mortality, length of stay), no formal imputation or adjustment will be performed for missing values. Analyses will be based on the available data at the time of analysis. If needed, descriptive statistics may be used to examine the extent and pattern of missing data.

- Summary of Missing Data Handling**:**
  - AI-SaMD score: Automatically imputed via internal LOCF logic within the system
  - Primary outcome data: Analyses will be based on observed data without imputation

### **2.4.3 Time Window**

Analyses of outcome variables will be limited to events occurring within predefined observation windows, calculated from a specified reference point (e.g., admission date, date of first alert). The observation window varies depending on the definition of each variable and is based on the predefined evaluation schedule (Table 1).

### **2.4.4 Unscheduled Visits**

As the AI-SaMD score is generated in real time in the ward setting, the concept of scheduled visits does not apply.

### **2.4.5 Centers Pooling**

This study involves four participating hospitals, each considered an independent cluster. No pooling of centers will be performed due to structural and operational differences among the hospitals. In statistical models, each hospital will be treated either as a fixed effect or as a cluster with appropriate correlation structure adjustments.

# **3. STATISTICAL ANALYSIS SOFTWARE**

All statistical analyses will be conducted using R (version 4.3 or higher) or Python (version 3.7 or higher). Both platforms are suitable for analyzing clustered and repeated measures data as in the SW-CRT design and support all planned models including GLMM.

All analytic code will be documented using standardized procedures, and analyses will be reproducible based on the methods outlined in this SAP. Key results and outputs will be stored in a verifiable format. The software used for each analysis (R or Python) will be explicitly stated in the final statistical report.

- Software used:
  - R version 4.3 or higher (lme4/glmer/glmmTMB/gee)
  - Python version 3.7 or higher (statsmodels/pymer4)

# **4. References**

1.	Escobar GJ, Liu VX, Schuler A, Lawson B, Greene JD, Kipnis P. Automated Identification of Adults at Risk for In-Hospital Clinical Deterioration. N Engl J Med. 2020;383(20):1951-60.

2.	DeVita MA, Hillman K, Bellomo R, Odell M, Jones DA, Winters BD, et al. Textbook of rapid response systems: concept and implementation: Springer; 2017.

3.	Cho KJ, Kim JS, Lee DH, Lee SM, Song MJ, Lim SY, et al. Prospective, multicenter validation of the deep learning-based cardiac arrest risk management system for predicting in-hospital cardiac arrest or unplanned intensive care unit transfer in patients admitted to general wards. Crit Care. 2023;27(1):346.

4.	Cho KJ, Kim KH, Choi J, Yoo D, Kim J. External Validation of Deep Learning-Based Cardiac Arrest Risk Management System for Predicting In-Hospital Cardiac Arrest in Patients Admitted to General Wards Based on Rapid Response System Operating and Nonoperating Periods: A Single-Center Study. Crit Care Med. 2024;52(3):e110-e20.

5.	Shin Y, Cho KJ, Lee Y, Choi YH, Jung JH, Kim SY, et al. Multicenter validation of a deep-learning-based pediatric early-warning system for prediction of deterioration events. Acute Crit Care. 2022;37(4):654-66.

6.	Lee YJ, Cho KJ, Kwon O, Park H, Lee Y, Kwon JM, et al. A multicentre validation study of the deep learning-based early warning score for predicting in-hospital cardiac arrest in patients admitted to general wards. Resuscitation. 2021;163:78-85.

7.	Kwon JM, Lee Y, Lee Y, Lee S, Park J. An Algorithm Based on Deep Learning for Predicting In-Hospital Cardiac Arrest. J Am Heart Assoc. 2018;7(13).

8.	Winslow CJ, Edelson DP, Churpek MM, Taneja M, Shah NS, Datta A, et al. The Impact of a Machine Learning Early Warning Score on Hospital Mortality: A Multicenter Clinical Intervention Trial. Crit Care Med. 2022;50(9):1339-47.

9.	Kollef MH, Chen Y, Heard K, LaRossa GN, Lu C, Martin NR, et al. A randomized trial of real-time automated clinical deterioration alerts sent to a rapid response team. J Hosp Med. 2014;9(7):424-9.

10.	Bailey TC, Chen Y, Mao Y, Lu C, Hackmann G, Micek ST, et al. A trial of a real-time alert for clinical deterioration in patients hospitalized on general medical wards. J Hosp Med. 2013;8(5):236-42.

11.	Adams R, Henry KE, Sridharan A, Soleimani H, Zhan A, Rawat N, et al. Prospective, multi-site study of patient outcomes after implementation of the TREWS machine learning-based early warning system for sepsis. Nat Med. 2022;28(7):1455-60.

12.	Henry KE, Adams R, Parent C, Soleimani H, Sridharan A, Johnson L, et al. Factors driving provider adoption of the TREWS machine learning-based early warning system and its effects on sepsis treatment timing. Nat Med. 2022;28(7):1447-54.

13.	Lin CS, Liu WT, Tsai DJ, Lou YS, Chang CH, Lee CC, et al. AI-enabled electrocardiography alert intervention and all-cause mortality: a pragmatic randomized clinical trial. Nat Med. 2024;30(5):1461-70.

14.	Maharaj R, Raffaele I, Wendon J. Rapid response systems: a systematic review and meta-analysis. Crit Care. 2015;19(1):254.

15.	Baio G, Copas A, Ambler G, Hargreaves J, Beard E, Omar RZ. Sample size calculation for a stepped wedge trial. Trials. 2015;16:354.

16.	Hussey MA, Hughes JP. Design and analysis of stepped wedge cluster randomized trials. Contemp Clin Trials. 2007;28(2):182-91.

17.	Li F, Hughes JP, Hemming K, Taljaard M, Melnick ER, Heagerty PJ. Mixed-effects models for the design and analysis of stepped wedge cluster randomized trials: An overview. Stat Methods Med Res. 2021;30(2):612-39.

18.	Kaplan EL, Meier P. Nonparametric estimation from incomplete observations. Journal of the American statistical association. 1958;53(282):457-81.

19.	Mantel N. Evaluation of survival data and two new rank order statistics arising in its consideration. Cancer Chemother Rep. 1966;50(3):163-70.

20.	Haukoos JS, Lewis RJ. The Propensity Score. JAMA. 2015;314(15):1637-8.
